# Specific recruitment of soil bacteria and fungi decomposers following a biostimulant application increased crop residues mineralization

**DOI:** 10.1371/journal.pone.0209089

**Published:** 2018-12-31

**Authors:** Eve Hellequin, Cécile Monard, Achim Quaiser, Morgane Henriot, Olivier Klarzynski, Françoise Binet

**Affiliations:** 1 University of Rennes, CNRS, ECOBIO [(Ecosystèmes, biodiversité, évolution)]—UMR 6553, Rennes, France; 2 BIO3G Company, Merdrignac, France; University of Porto, PORTUGAL

## Abstract

Agriculture is undergoing important changes in order to meet sustainable soil management with respect to biodiversity (namely agroecology). Within this context, alternative solutions to mineral fertilizers such as agricultural biostimulants are thus promoted and being developed. The mechanisms by which some soil biostimulants sustain soil biological functioning and indirectly increase crop yields are still unknown. Our goal in the present study was to demonstrate if and to what extent the application of a soil biostimulant affects the soil heterotrophic microbial communities that are involved in organic matter decomposition and carbon mineralization. We hypothesized that the addition of a biostimulant results in changes in the composition and in the biomass of soil microbial communities. This in turn increases the mineralization of the organic matter derived from crop residues. We performed soil microcosm experiments with the addition of crop residues and a biostimulant, and we monitored the organic carbon (orgC) mineralization and the microbial biomass, along with the microbial community composition by sequencing 16S rRNA gene and ITS amplicons. The addition of a soil biostimulant caused a pH neutralizing effect and simultaneous enhancement of the orgC mineralization of crop residues (+ 400 μg orgC g^-1^ dry soil) and microbial biomass (+ 60 μg orgC g^-1^ dry soil) that were linked to changes in the soil microbial communities. Our findings suggest that the soil carbon mineralization enhancement in the presence of the biostimulant was supported by the specific recruitment of soil bacteria and fungi. Whereas archaea remained stable, several operational taxonomic units (OTUs) of indigenous soil bacteria and fungi were enriched and affiliated with known microbial decomposers such as *Cytophagaceae*, *Phaselicystis* sp., *Verrucomicrobia*, *Pseudomonas* sp., *Ramicandelaber* sp., and *Mortierella* sp., resulting in lower soil microbial richness and diversity.

## 1. Introduction

Concurrently with the ecological transition, agricultural practices attempt to reduce their dependence on chemicals and to sustain natural resources while producing healthy food. The field of agroecology is developing and is looking for integrative crop management systems that take the ecological processes sustained by the high biodiversity found in soils into account [1]. Soil plays an essential role in the functioning of terrestrial ecosystems and soil microorganisms are involved in up to 90% of the ongoing processes [2]. Microorganisms are involved in various biogeochemical cycles and therefore contribute to soil fertility [3,4], providing mineral nutrients to crops through the mineralization of soil organic matter (SOM) [5]. Because this ecosystem service of SOM mineralization relies mainly on bacteria, fungi and archaea [6], changes in either their diversity, abundance or activity may significantly impact the subsequent supply of nutrients to plants. The manipulation of soil microbiota has thus emerged as a new practice in agriculture with respect to the ecological transition that promote sustainable soils. The use of agricultural biostimulants (BS) that are intended to stimulate and regulate soil microorganisms could be promoted as an alternative solution to the use of mineral fertilizers, which are more expensive [7–9].

According to the European Biostimulant Industry Council, BS can either be applied directly on the plants to favor their growth and development or on the soil in order to induce changes in its physico-chemical and microbial properties, and indirectly enhance plant functioning [10]. While most of the available studies focused on the direct beneficial effects of BS applied on plants [11,12], knowledge about the impact of BS on soil is limited and the mechanisms improving soil biological functioning and crop yields are still misunderstood. Among the various biostimulant products applied on the soil, different effects can be observed, such as changes in the soil physico-chemical properties that may either stimulate the growth of beneficial fungi [13], reduce the development of pathogen organisms [14], or increase the microbial activity by providing compounds directly used by soil microorganisms and/or plants such as peptides, amino acids, polysaccharides, humic acids and phytohormones [15,16]. However, there is still a clear need to identify their way of action to optimize their formulations and uses.

To improve our understanding about the effects of soil BS input on the soil biota, we focused our research on a soil inoculant formulation (namely, soil biostimulant (BS) throughout the text), intended to be applied directly on crop residues in agricultural soils to improve their decay and mineralization, thereby preserving the fertility of the soil. Several studies on crop residue management have indicated that the decomposition process was strongly influenced by the quality (e.g. type of residue, biochemical composition), or placement of residues in the soil depending on tillage practices (e.g. surface/incorporated) [17–19]. Thereby, in the current context of reducing the input of mineral fertilizers, crop residue management is important in order to ensure sustainable crop production and to provide a constant pool of nutrients for the crops.

In the present study, our aim was to determine the responses of soil bacteria, fungi and archaea to the addition of the BS in the soil. Our objectives were to (i) quantify the effect of BS addition on the soil microbial biomass and OM mineralization, (ii) determine changes in the composition of the soil microbial communities (iii) identify whether microbial changes concern indigenous soil microorganisms or specific microorganisms that are naturally present in the BS. We hypothesized that changes in both the composition and biomass of the microbial communities induced by the BS will increase the mineralization of the OM derived from crop residues. Using soil microcosm incubations, we monitored the microbial biomass carbon as well as the organic carbon (orgC) mineralization and we analyzed the changes in microbial diversity following the addition of a BS by using a 16S rRNA gene and ITS amplicon sequencing approach. We demonstrated in this study that an agricultural soil BS increased the orgC mineralization and induced subtle changes in the composition of the microbial communities, in particular by sustaining certain microbial decomposers.

## 2. Materials and methods

### 2.1 Soil and biostimulant characteristics

Topsoil (0–20 cm depth) that was used as starting material for conducting the lab incubation experiment was collected on a private agricultural site dedicated to experimental studies (the site of La Jaillière, France, 47°27'06.3"N/0°57'58.4"W) for which no specific permission was required. Also, this study did not involve endangered or protected species. The soil was analyzed for its physico-chemical characteristics according to Carter and Gregorich [20] and had a pH_water_ of 6.7, a texture composed of 57% silt, 26.3% sand and 15% clay and it contained 1.7% of OM (i.e. 0.99% orgC) and 0.09% of total nitrogen (Ntot). The biostimulant developed and provided for free by the company BIO3G is in solid form, composed of natural raw materials without any additives, and is intended for application on crop residues before they are buried in the soil. It had a pH_water_ of 6.25 ± 0.0845 and it contained 35% of OM (i.e. 20.4% orgC) and 2.15% of total N as well as several labile organic compounds (low-weight molecules) such as amino acids. More information on the composition of the BS is given in Table 1.

**Table 1:** Analytical composition of the biostimulant under study.

	Content (g/100 g dry BS)
**Dry extract**	92
**Humidity**	8
**Raw ashes**	56.70
**Organic matter**	35
**Proteins**	13.40
**Total nitrogen**	2.15
**Phosphorus**	0.3
**Calcium**	15
**Sulfur**	2.1
**Magnesium**	0.86
**Amino acids**	
Alanine	1.20
Arginine	0.54
Aspartic acid	1.09
Cysteine	0.25
Glutamic acid	1.90
Glycine	0.66
Histidine	0.37
Hydroxyproline	<0.05
Isoleucine	0.55
Leucine	1.32
Lysine	0.68
Methionine	0.28
Ornithine	<0.05
Phenylalanine	0.59
Proline	1.08
Serine	0.73
Threonine	0.62
Total tryptophane	0.10
Tyrosine	0.42
Valine	0.76

### 2.2 Experimental design and soil microcosm incubation

The air dried soil was adjusted to 60% of the water hold capacity (i.e. 18%) and sieved (mesh size 4 mm) prior to soil microcosm incubations, according to the AFNOR standard (AFNOR XP U 44–163). Each soil microcosm contained the equivalent of 25 g of dry soil placed in hermetically closed 1 L glass jars to allow the CO_2_ produced to accumulate in the headspace; the air of the headspace was entirely renewed each time a measurement was taken (once a week). The soil microcosms were subjected to three different treatments each in three replicates: i) soil alone as a control (CS), ii) soil mixed with 120 mg of straw (SS) simulating 4.8 kg of wheat residues per m^2^ and corresponding to an input of 50 mg of orgC and 0.94 mg of total N, and iii) soil mixed with 120 mg of straw and the BS (SBS). This last treatment was prepared by applying 100 mg of the BS to 25 g of straw, and 120 mg of this mixture was then incorporated in the soil. In the SBS treatment, the additional orgC and total N amounts due to the BS input were negligible (100 μg and 10 μg, respectively) compared to both the orgC and total N amounts due to the straw input and the initial contents of the soil (245 mg orgC and 22.5 mg total N per microcosm, respectively). The initial raw materials (soil, straw and BS at t = 0) and the three soil microcosm treatments (CS, SS and SBS), each performed in three replicates, were characterized for their soil OM, orgC and total N contents, as well as for their microbial biomass and pH_water_ (Table 2). Soil microcosms were equipped with a NaOH trap to collect the evolved CO_2_ and incubated in the dark for 49 days at 28°C. Soil incubations were performed for seven weeks (49 days). After 3, 7, 14, 21, 28, 42 and 49 days of incubation, the CO_2_ trapped in 10 ml of 0.5 N NaOH was quantified by titration with 0.1 N HCl according to the AFNOR standard (AFNOR XP U 44–163) and the soil moisture was maintained by replacing the weight loss with sterile water. At the end of incubation, the pH of the soil microcosms were measured in water with a pH meter using a soil-to-water ratio of 1:5 (Table 2) and the soil samples were stored at -20°C for further chemical and microbial analyses. The soil microbial communities were analyzed after seven weeks of incubation, when the communities of microbial decomposers were presumed to be well established and stable in each soil treatment.

**Table 2:** Organic matter (OM), organic carbon (orgC) and total nitrogen (Ntot) concentrations in the original soil, straw and biostimulant and total contents in the soil microcosms at the beginning of incubation.

	Organic matter (mg.g^-1^ d.w)	Organic carbon (mg.g^-1^d.w)	Total nitrogen (mg.g^-1^ d.w)	Microbial Biomass (μgC.g^-1^ d.w)	pH_water_
**Raw materials**					
soil	17.0	9.9	0.9	140.4	6.7
straw	860.4	430.2	7.8	0*	N.D
BS	350.0	204.0	21.5	0*	6.3
**Soil microcosms**	(*mg per 25g of dry soil)*			*(*μ*gC*.*g*^*-1*^*d*.*w)*	
control soil (CS)	425	245.0	22.5	N.D	6.0
soil with straw (SS)	528.3	295.0	23.4	141.4	6.3
soil with straw and BS(SBS)	528.4	295.1	23.5	201.4	6.9

The whole measurements are expressed on a soil dry weight basis (d.w).

*Negatives values for the microbial biomass carbon were obtained for BS and straw and are explained in the materials and methods section (2.2). ND = Not Determined.

### 2.3 Microbial carbon biomass

The chloroform fumigation and extraction method described by Vance et al. [21] was used to measure the soil microbial biomass carbon in the initial raw materials and for all of the SS and SBS soil samples, after 49 days of incubation by using a Total Organic Carbon analyzer (Bioritech, Voisins-le-Bretonneux, France). The microbial biomass carbon was calculated using the equation of Vance et al. [21]:

BiomassC=(Cf−Cnf)×Kc (1)

where *Cf* is the dissolved organic carbon in fumigated soil (or straw or biostimulant), *Cnf* is the dissolved organic carbon in non-fumigated soil (or straw or biostimulant) and *Kc* is the correction factor of 2.64.

We obtained negative microbial biomass carbon values for the BS and straw samples; a possible explanation for this is that the fumigation method is not suitable for determining the microbial biomass in these materials or possibly because the % of dry matter for the straw and BS was close to 93% for both and it is well known that microbial activity is low in dry matter.

### 2.4 Soil and BS microbial communities: DNA extraction and library construction

DNA was extracted from 3 g of either soil or the BS using a protocol adapted from Quaiser et al. [22] with 25 mL of lysis buffer (Cetyltrimethylammonium bromide (CTAB) 4%, Polyvinylpyrrolidone (PVP) 0.5%, NaCl 0.75 M, potassium-phosphate 100 mM (50:50—K_2_HPO_4_:KH_2_PO_4_), Ethylenediaminetetraacetic acid (EDTA) 20 mM, β-mercaptoethanol 1%, guanidine thiocyanate 1 M) preheated to 65°C and from 1 g of the BS using 7 ml of lysis buffer. After 30 min at 65°C and intermittent vortexing every 5 min, one volume of chloroform-isoamylalcohol (24:1) was added, the samples were mixed by vortexing for 1 min and centrifuged during 30 min at 4500 rpm. The aqueous phase was recovered and the DNA was precipitated by adding 0.5 volume of pure ethanol or 1 volume of 30% polyethylene glycol (PEG) for the BS samples. The DNA extracts were purified using the NucleoSpin gDNA Cleanup kit (Macherey-Nagel) according to the manufacturers’ instructions. The genomic DNA quality was assessed on 1% agarose gel, quantified on a nanodrop spectrophotometer (ND-1000, Nyxor Biotech, Palaiseau, France) and quickly stored at -20°C.

The bacterial and archaeal 16S rRNA gene libraries and the fungal ITS libraries were constructed using the "16S metagenomics sequencing library preparation" protocol given by Illumina (https://www.illumina.com/content/dam/illumina-support/documents/documentation/chemistry_documentation/16s/16s-metagenomic-library-prep-guide-15044223-b.pdf) [23] which is a two-step polymerase chain reaction (PCR) approach. The following primer sets were used for the bacteria, fungi and archaea: 341F (5'-CCTACGGGNGGCWGCAG-3') and 785R (5'-GACTACHVGGGTATCTAATCC-3') [24], ITS1f (5'-CTTGGTCATTTAGAGGAAGTAA-3') [25] and ITS2 (5'-GCTGCGTTCTTCATCGATGC-3') [26] and Arch344f (5'-ACGGGGYGCAGCAGGCGCGA-3') [27] and Arch806R (5'-GGACTACVSGGGTATCTAAT-3') [28], respectively. Each primer set contains the overhang adapter: forward overhang (5'-TCGTCGGCAGCGTCAGATGTGTATAAGAGACAG-3') and reverse overhang (5'-GTCTCGTGGGCTCGGAGATGTGTATAAGAGACAG-3') and targets the variable V3 and V4 regions of the 16S rRNA gene for the bacterial and archaeal ones and the ITS1 region for fungal ones. PCRs were conducted in a final volume of 25 μl containing each bacterial or fungal primer (0.2 μM), each archaeal primer (0.6 μM), 12.5 μl 2X KAPA HiFiHotStart Ready Mix (2X), 2 μl of DNA and ultrapure water to reach the final volume. The amplification conditions were as follows: for bacteria, 3 min at 95°C, 25 cycles of 30 s at 95°C, 30 s at 55°C and 30 s at 72°C and a final 5 min extension step at 72°C; for archaea, 5 min at 95°C, followed by 30 cycles of 20 s at 98°C, 15 s at 66°C and 15 s at 72°C and a final 1 min extension step at 72°C; for fungi, 4 min at 95°C, followed by 30 cycles of 30 s at 95°C, 30 s at 56°C and 30 s at 72°C and a final 10 min extension step at 72°C. Two independent PCR replicates were performed for each sample, the PCR products were pooled and purified using the Agencourt AMPure XP beads system. The second PCR reaction attached specific indexes (i5, i7) to identify each sample and to the Illumina sequencing adapters (P5, P7) using the Nextera XT index kit. This was performed in a final volume of 50 μl containing 5 μl of each Nextera XT index primer, 5 μl of the first PCR product, 25 μl of the KAPA HiFiHotStart Ready Mix (2X) and 10 μl of PCR grade water. The amplification conditions consisted of 3 min at 95°C, followed by 8 cycles of 30 s at 95°C, 30 s at 55°C and 30 s at 72°C, and a final 5 min extension step at 72°C. The PCR products were purified with the Agencourt AMPure XP beads system.

The amplified bacterial, fungal and archaeal products were quantified by qPCR (light cycler 480, Roche, Meylan, France). Each well contained 3 μl of SYBR Green PCR Master Mix, 3 μl of the PCR product and Illumina primers 0.2 μM final P5 (5’-AATGATACGGCGACCACCGA-3') and P7 (5’-CAAGCAGAAGACGGCATACGA-3'). The qPCR program consisted of 3 min at 95°C, followed by 45 cycles of 30 s at 95°C, 45 s at 60°C, 20 s at 72°C and a final melting curve step of 0.05 s at 95°C, 1 min at 65°C and an increase of 0.06°C/s from 65°C to 97°C. Then, the amplified products were combined in a unique pool in an equimolar ratio and sequenced using 2x250 bp paired-end Illumina MiSeq with 20% PhiX at the “Human and Environmental Genomic” platform (Rennes, France).

### 2.5 Microbial sequence analysis

For all of the samples (raw material and soil microcosms), which were performed each in three replicates, we obtained the following total number of raw reads: 1,325,903 for bacteria, 1,955,564 for archaea and 2,607,580 for fungi. These sequences are available on the sequence read archive (SRA) database (Bioproject SRP104693). The sequence read quality was controlled with FastQC [29]. The bacterial and archaeal analyses were performed using the FROGS pipeline [30] and the PIPITS pipeline was used for the fungal analyses [31].

Applying the FROGS pipeline, the reads were merged with a minimum overlap of 20 bp (FLASH) [32], filtered according to the following criteria: expected amplicon size of 480 bp for bacteria and 460 bp for archaea, minimal length of 450 bp for bacteria and 420 bp for archaea, and maximal length of 480 bp for both and no ambiguous nucleotides were allowed. The primer sequences and sequences where the two primers were not present were removed and the sequences were dereplicated. The sequences were clustered using the swarm method [33] with an aggregation distance equal to 3 for clustering. The chimera were removed using the VSEARCH tool with the UCHIME de novo method [34,35] combined with a cross-sample validation. The taxonomy affiliation was performed using the SILVA database (Silva 128) [36].

For fungi, the first step of the PIPITS pipeline was to the merge reads with a minimum overlap of 20 bp, a minimal length of 250 bp and a maximal length of 480 bp. The assembled reads were then filtered with a quality score of 33. The second step consisted of dereplicating the sequences and extracting the ITS1 subregion. The dereplicated sequences were clustered at a threshold similarity of 97%. The chimera were removed using UCHIME in UNITE [34] and the representative operational taxonomic units (OTUs) were taxonomically assigned using the RDP classifier compared against the Warcup fungal ITS reference database.

The bacterial, archaeal and fungal OTUs present in at least 3 out of 15 samples and representing at least 0.0005% of all sequences were retained for the subsequent analyses. Thus, the bacterial OTUs were represented by a minimum of 6 sequences, and there were 8 sequences for the archaeal OTUs and 10 for the fungal OTUs. See S1 Table for more details on the data processing.

### 2.6 Data analysis

All of the statistical analyses were performed using R (v3.3.2, Core Team, 2016). To avoid sampling size effects, the number of reads per sample was randomly subsampled to the lowest number of reads; the soil dataset was normalized separately from the BS and straw dataset: 51 829 reads for bacteria, 26 669 for fungi and 73 142 for archaea in the soil samples and 42 473 for bacteria, 102 839 for fungi and 4842 for archaea in the BS and straw samples. According to the rarefaction curves, the sequencing effort provided a good estimate of the bacterial, fungal and archaeal richness in the soil samples (S1, S2 and S3 Figs). The Shannon diversity index was used to estimate the alpha diversity and the richness was estimated as the number of OTUs. A Student’s t-test was used to test for differences in the microbial biomass carbon between the soil treatments, and non-parametric Kruskal-Wallis analyses were performed on the amount of orgC mineralized, the Shannon diversity indices and the observed richness values. A powered partial least squares discriminant analysis (PPLS-DA) was generated at the OTU level to compare the bacterial, fungal and archaeal community compositions when straw and/or the BS were applied to the soil. The individual plots coupled with a statistical permutation test based on a cross-model validation were used to identify how the groups, represented by the various treatments, were structured. An analysis of variance (ANOVA) was carried out on each OTU to identify significant differences in their abundances in the presence of the BS. Only OTUs present in the three replicates of each soil treatment (S2 and S3 Tables) were retained for further analysis. A BLASTn analysis [37] of the representative sequences for the enriched OTUs was performed to test their similarity to sequences from microorganisms native to the soil environment.

## 3. Results and discussion

### 3.1 Soil carbon content and mineralization

**Fig 1:**
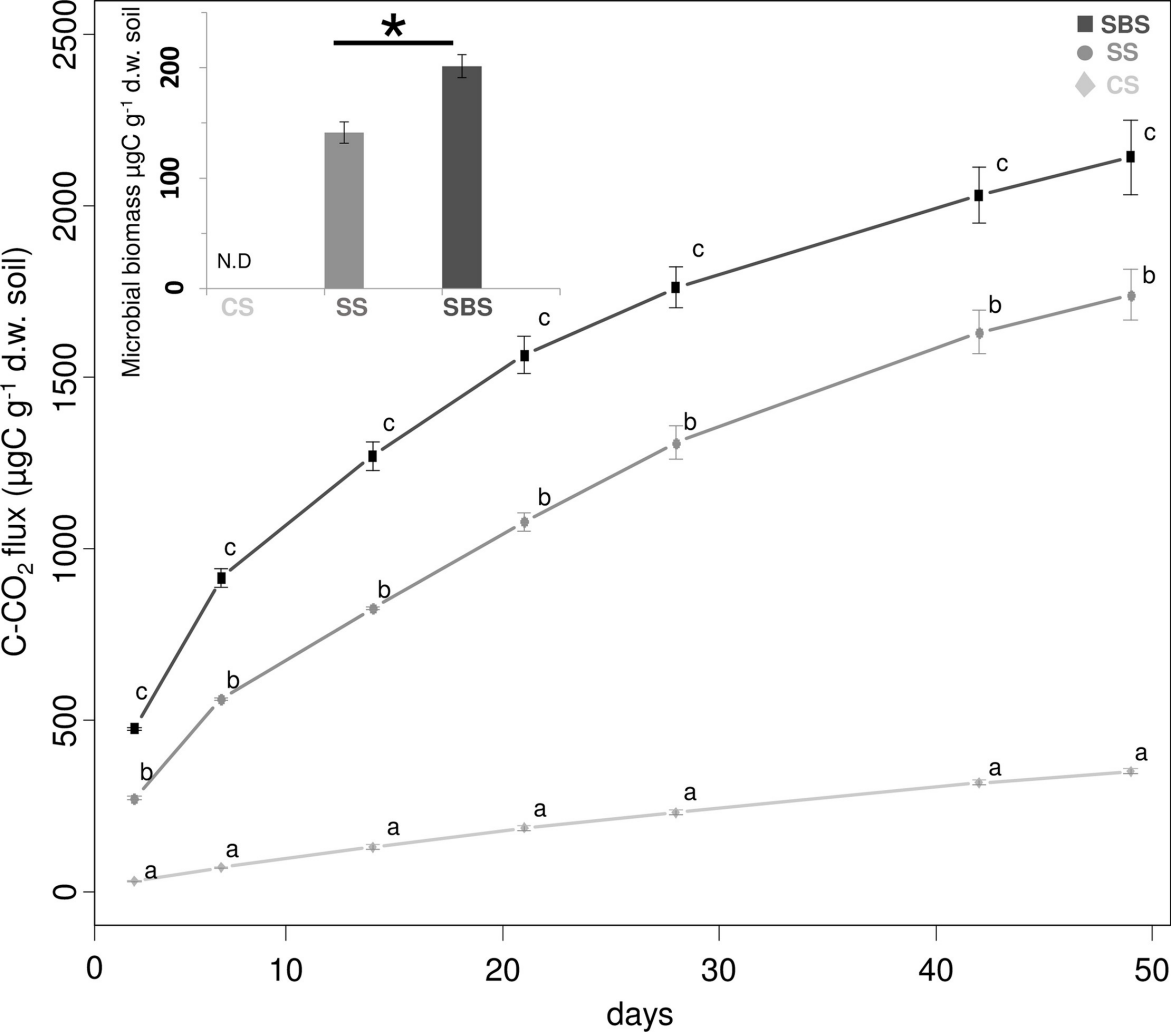
Cumulative kinetics of the orgC mineralization and microbial biomass carbon in, the control soil (CS), the soil with straw (SS) and the soil with straw and the BS (SBS). The error bars indicate the standard errors of the C-CO_2_ emission mean values (n = 3). At each sampling date, the data indicated with different letters are significantly different according to the Kruskal-Wallis test. The microbial biomass carbon in the control soil was not determined (ND). The C-CO_2_ emission and microbial biomass are “expressed” per g of dry soil (d.w.). The (*) correspond to significantly different values of microbial biomass carbon.

After 49 days of incubation, a total of 350 ± 8, 1740 ± 70 and 2140 ± 110 μg g^-1^ dry soil C-CO_2_ was emitted in the CS, SS and SBS treatments, respectively (Fig 1). In the SS treatment, we observed a significant increase in emitted CO_2_ compared to the CS treatment indicating that the straw, as an added organic substrate, was metabolized by heterotrophic microorganisms. The increased emission of CO_2_ observed in the SS compared to the CS treatments (+1390 μg of C-CO_2_) (Kruskal-Wallis, H = 7.2, *P* = 0.027) corresponded to 69.5% of the orgC potentially derived from the inoculated straw. In the presence of the BS, the mineralization was statistically higher in SBS than in SS (2140 ± 110 versus 1740 ± 70 μg g^-1^) leading to a net increase of an additional 400 μg of C mineralized per gram of soil, indicating that potentially 89.5% of the orgC of the inoculated straw was mineralized. To confirm this, future experiments should be carried out using ^13^C labelled straw so as to distinguish between soil-derived mineralized C and straw-derived mineralized C. The increase in orgC mineralization in the SBS treatment compared to the SS treatment was linked to a significantly higher microbial biomass (201.4 ± 10.4 μg C g^-1^ dry soil in SBS and 141.4 ± 9 μg C g^-1^ dry soil in SS) (*t*-test, *P* = 0.0018). The C microbial biomass values obtained for the SS treatment were in agreement with another study that reported values ranging from 67 μg C g^-1^ dry soil to 166 μg C g^-1^ dry soil in 13 agricultural soils [38], while the C microbial biomass of the SBS treatment was closer to that observed in forest soils (215 μg C g^-1^ dry soil) but lower than those in grassland soils (250 μg C g^-1^ dry soil) [39].

As hypothesized, mineralization and microbial biomass were increased in the presence of the BS. These results are in agreement with the results of other studies with regards to the effect of other BS on soil microorganisms. Chen et al. [40,41] demonstrated that soil amendments using two BS stimulated the microbial activity and increased the decomposition rates of straw more than two-fold compared to that in the control soil, as well as the mineralization of soil OM. Another study by Tejada et al. [42] demonstrated that four BS applied on the soil, which differed in terms of their chemical composition, enhanced the soil enzymatic activities and increased both the fungal and bacterial biomass by two or three fold, depending on the BS.

The increases in the C-CO_2_ emissions and the enhanced microbial biomass storage in SBS compared to SS corresponded to a total of 460 μg orgC g^-1^ dry soil that could not be attributed to the addition of orgC contained in the BS itself since it only corresponded to an input of 4 μg of orgC g^-1^ dry soil. Similarly, this stimulation of the microbial activity could not be due to soil N enrichment coming from the BS since it only corresponded to an input of 0.41 μg of total N per gram of dry soil which was negligible compared to the initial content in the soil (Table 1). Tejada et al. [42] highlighted a better stimulation of soil enzymatic activities in BS-amended soil than with other sources of organic matter applied to the soil. As suggested by Parrado et al. [43], the higher stimulation of the soil microbial community may have been due to the application of a BS with high contents of low molecular weight proteins that can be directly assimilated by the soil microorganisms. Our study showed that the BS used significantly increased the OM mineralization and microbial biomass, without adding a significant amount of orgC or total N. It is possible that the BS may have supplied proteins with low molecular weight, amino acids [44] or growth factors such as phytohormones [45] that stimulated the microbial community, although these factors have not been studied here. However as we can see in Table 1, amino acids are widely present in the BS and they are used in protein synthesis or can be directly absorbed by the soil microorganisms as an alternative source of nitrogen and carbon [46]. It has also have been reported that they enhance soil respiration and microbial biomass activity [47].

Furthermore, it is well documented that the incorporation of fresh OM in soil may increase SOM degradation [48,49]. For example, by using a ^13^C labeled wheat residue, Pascault et al. [50] demonstrated a higher release of ^12^CO_2_ with wheat residues compared to the non-amended control soil, pointing to a priming effect of native unlabeled SOM. The changes in the native SOM degradation as a result of an exogenous substrate depends on the substrate quality [51]. The input of crop residues that are known to decompose slowly result in a rapid response from the microorganisms by producing enzymes that are able to degrade this exogenous organic matter [52]; hence, microorganisms are also able to decompose SOM [53]. Other studies using low molecular weight substances such as glucose or amino acids also demonstrated the occurrence of a priming effect [54,55] and this type of molecules was present in the BS. This suggests that, in our study, the addition of straw in the SS and SBS treatments may have promoted this priming effect. However, we could not confirm that the increase in soil orgC mineralization we observed was due to the degradation of the straw itself or to the occurrence of a priming effect, with a higher intensity in the presence of the BS.

### 3.2 Microbial community composition

**Fig 2:**
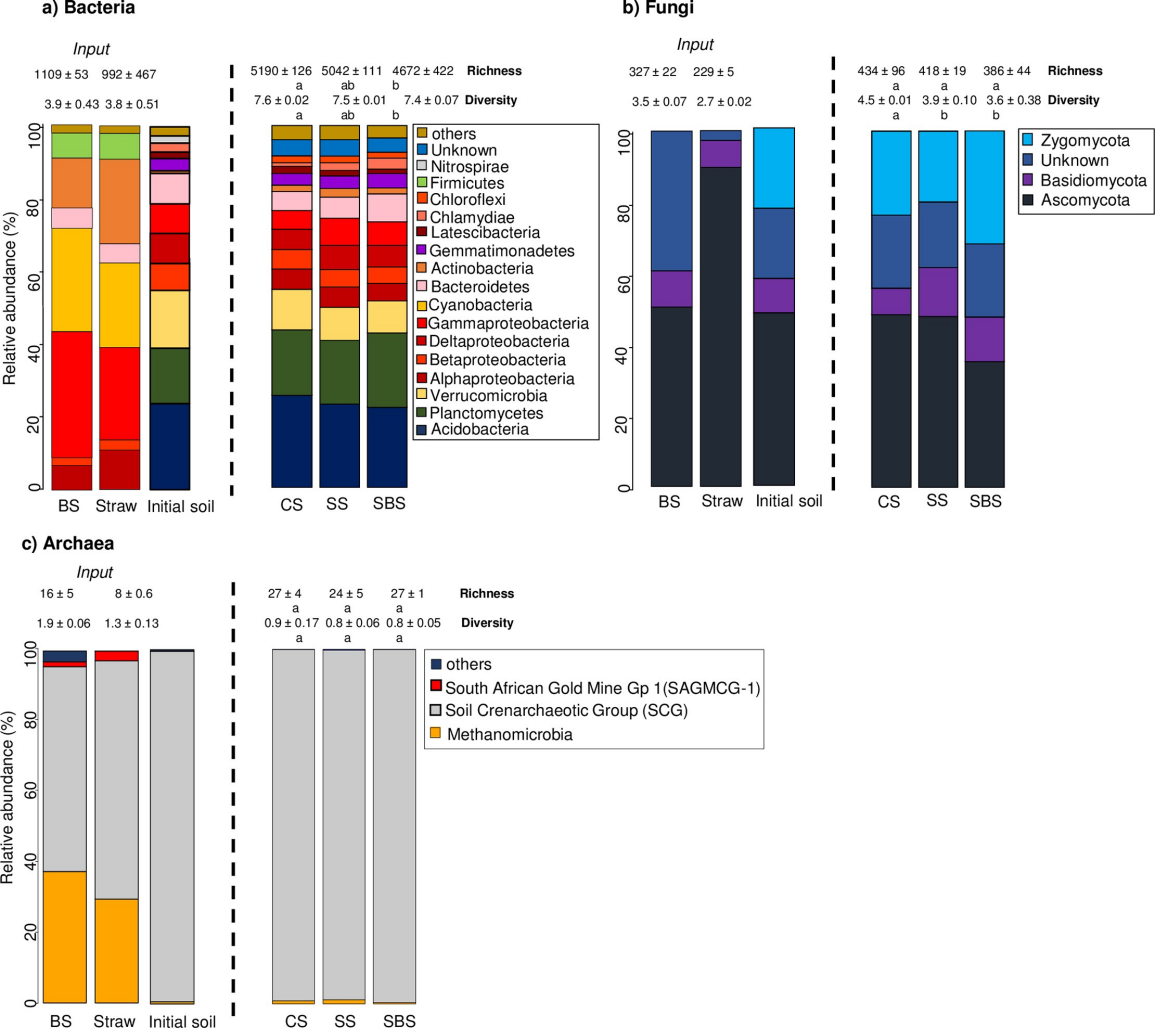
**Composition of the bacterial (a), fungal (b) and archaeal (c) microbial community in the various samples. Pristine microbial composition of the BS and straw (“Input”) and composition of the bacterial, fungal and archaeal communities at the phylum level (and class level for the *Proteobacteria* and archaea) in the three soil treatments after 7 weeks (49 days) of incubation with the associated richness and Shannon diversity index.** BS: biostimulant samples, CS: control soil, SS: soil with straw, SBS: soil with straw and BS, Means and standard errors were calculated (n = 3). The Kruskal-Wallis test was performed (*P*<0.05). Different letters correspond to significantly different values.

The simultaneous enhancement of the orgC mineralization and microbial biomass were linked to changes in the soil microbial communities. These changes in the microbial community were not related to the pristine microbial composition of the BS itself (Fig 2). The main native bacterial and fungal phyla that characterized the BS, such as *Proteobacteria* (*β-Proteobacteria)*, *Cyanobacteria*, *Actinobacteria* and *Ascomycota*, were not more represented in the SBS than in the CS and SS treatments. For example, two bacterial OTUs were highly dominant in the BS (*Cyanobacteria*, OTU1, and *γ-Proteobacteria*, OTU3, representing 26.7% and 10.3% of the total sequences, respectively) but were detected at very low abundances in the SBS sample (0.01% and 0.04% of the total sequences, respectively). For archaea, among the three classes detected in the BS (the Soil crenarchaotic group (SCG), *Methanomicrobia* and South African gold mine group), only SCG was predominant in the three soil treatments. Therefore, our results showed that the enhanced mineralization observed in the SBS treatment was not related to a bioaugmentation through the inoculation of specific microorganisms present in the BS.

Simultaneously, the BS may have exerted an indirect effect on the microbial community by altering the pH of the soil. While the pH of the BS was 6.25, the pH values was significantly higher up to one half-unit pH in the SBS treatment (6.87 ± 0.15 versus 6.33 ± 0.13 in the SBS and CS treatments, respectively; ANOVA p-value = 4.67.10^−9^) (Table 2). The soil pH is one of the most influential factors affecting the microbial community in soil [56] as it influences abiotic factors such as carbon availability [57] or biotic factors such as the composition of fungal and bacterial biomass [58] in agricultural soils. The soil can be subdivided into acidic (pH<5), moderately acidic (pH = 5–6.5), neutral (pH = 6.6–7.5), and alkaline soils (pH > 7.5) [59]. Therefore, our results showed that the BS had a pH neutralizing effect that may have induced changes in the bacterial and fungal communities.

#### 3.2.1 Bacterial phyla

Irrespective of which soil treatment was used, the same phyla (e.g. *Acidobacteria*, *Planctomycetes* and *Proteobacteria*) dominated the soil bacterial communities. However, even at the phyla level, several changes were observed in the presence of the BS (SBS) compared to the soil amended with straw (SS): the proportion of *Planctomycetes* was significantly higher from 17.6% to 20.5%, and the trend for the proportion of *Bacteroidetes* and *Chlamydiae* was also higher, but non-significantly, from 5.8% to 7.7% and from 2.16% to 2.9%, respectively (Fig 2A). At the same time, the proportion of *γ-Proteobacteria* tended to be lower in the SBS treatment than in SS (6.4% and 7.5%, respectively). Previous studies have reported that the abundance of *Bacteroidetes* was highest in soils with high C availability and was positively correlated with the C mineralization rates [60] and that active *Bacteroidetes* members were some of the initial metabolizers of the labile carbon inputs [61]. These are consistent with our observation, the highest abundance of *Bacteroidetes* at 49 days being probably linked to the use of labile C in the soil microcosm during the course of the incubation. In addition, the soil pH could have played a role in these bacterial community changes in the SBS treatment. We showed that the abundance of *Bacteroidetes* was higher in soil with higher pH, which is in agreement with Lauber et al. [62] who demonstrated a significant and positive correlation between *Bacteroidetes* abundance and soil pH (for pH values ranging from 4 to 8). At the opposite, our findings on *Planctomycetes*, were inconsistent with the recent report of Zhang et al. [63]. Whereas the latter authors showed a decrease in the *Planctomycetes* abundance from acidic to near-neutral pH values and then an increase from near-neutral to alkaline pH values, we demonstrated an increase from moderately acidic to near-neutral pH values.

#### 3.2.2 Fungal phyla

The addition of the BS induced greater changes in the composition of the fungal phyla since the relative abundances of *Zygomycota* was significantly higher in SS than in SBS from 19.7% to 31.6% and *Ascomycota* tended to be lower from 47.9% to 35.3%, respectively (Fig 2B). Generally, fungi grow well and tolerate acidic soils better than bacteria [64], but some fungi belonging to phylum of *Zygomycota* and saprotrophic species (e.g. *Amblyosporium*, *Pseudombrophila*, *Coprinus*, *Mortierella*) have been shown to grow well in neutral to slightly alkaline conditions [65–67].These findings are consistent with our results and suggest that the addition of the BS induced changes in fungal communities through its pH neutralizing effect. Moreover, with the amendment of the straw by itself, the proportion of *Basidiomycota* tended to be higher in both the SS and SBS treatments compared to the CS treatment resulting in a lower fungal diversity in these treatments (Fig 2B). As a result, the straw input may have selected *Basidiomycota* which are known litter decomposers able to degrade complex and recalcitrant OM [68,69].

#### 3.2.3 Archaeal phyla

Significant changes were not seen in the archaeal community in the presence of the BS. At the class level, 99% of the archaeal sequences were affiliated with the Soil Crenarchaeotic Group (SCG) in all of the soil treatments. The SCG class falls within the recently described *Thaumarchaeota* phylum; these archaea are thought to be chemolithoautotrophs that use ammonium as an energy source [70]. To date, there is no proof that organisms belonging to this phylum play a role in orgC mineralization [6]. So far, it appears that *Thaumarchaeota* are directly involved in nitrogen metabolism and mainly comprise ammonia-oxidizing archaea [71], which is in line with our results where no changes in the community structure of archaea was observed when straw and the BS were added. Furthermore, a study of Hu et al. [72] demonstrated that *Thaumarchaeota* represented more than 85% of the total archaea in soils with pH higher than 6, which is in agreement with our results: the three soils microcosms are dominated by the *Thaumarcheaota* and have pH values ranging from 6 to 6.9.

### 3.3 Microbial diversity and richness

In order to determine the potential changes in the microbial communities due to the amendments, we compared the richness and diversity of the microbial communities using 16S/ITS1 rRNA amplicons (Fig 2). Statistical differences (*P* < 0.05) were observed between the CS and SBS treatments in terms of bacterial richness and the bacterial and fungal Shannon diversity index, as well as between the CS and SS treatments for the fungal Shannon diversity index. Because the bacterial and fungal diversities and bacterial richness were not different between CS and SS treatment but significantly lowest in the SBS compared to the CS, this suggested that this lowest diversities and richness could have been induced by a synergic effect of the BS and straw which thereby established a dominance of certain bacteria and fungi. After 49 days of incubations, it is probable that easily degradable compounds gradually gave way to recalcitrant compounds that were more difficult to degrade and which therefore required specific enzymes to do so [52]. Hence, the lowest bacterial and fungal diversity due to the addition of straw and the BS can be explained by the degradation of more recalcitrant compounds that can be carried out by a few groups of organisms with specific functions such as the production of extracellular enzymes [73]. Therefore, due to the soil microbial enzymatic specificity for substrate degradation, a succession of microorganisms is observed throughout the decomposition of the OM [74]. However, further temporal dynamics experiments should be undertaken to clearly determine the microbial community succession in our study.

Among the bacterial and fungal phyla enhanced in the presence of the BS after 49 days of incubation, different functional strategies regarding the use of soil carbon can be identified according to the ecological classification scheme suggested by Fierer et al [60]. *Planctomycetes* are described as a phylum that can exhibit oligotrophic tendencies with slow growth and a K-selected life strategy [75], and *Bacteroidetes* are mainly copiotrophs [60]. Among the changes detected in the fungal phyla, *Zygomycota* are known to use readily available sugars and to be the first fungi to colonize a fresh substrate, but are also able to degrade recalcitrant compounds [76,77]. Therefore, at the phylum level, the application of the BS on straw residues sustained several microbial phyla but without any specific functional strategy towards OM decomposition optimization with either oligotrophic or copiotrophic tendencies. Also, we may consider that 49 days of incubation was not long enough to allow microbial successions and the development of specific functional strategies in the soil microbial communities.

### 3.4 Microbial recruitment

**Fig 3:**
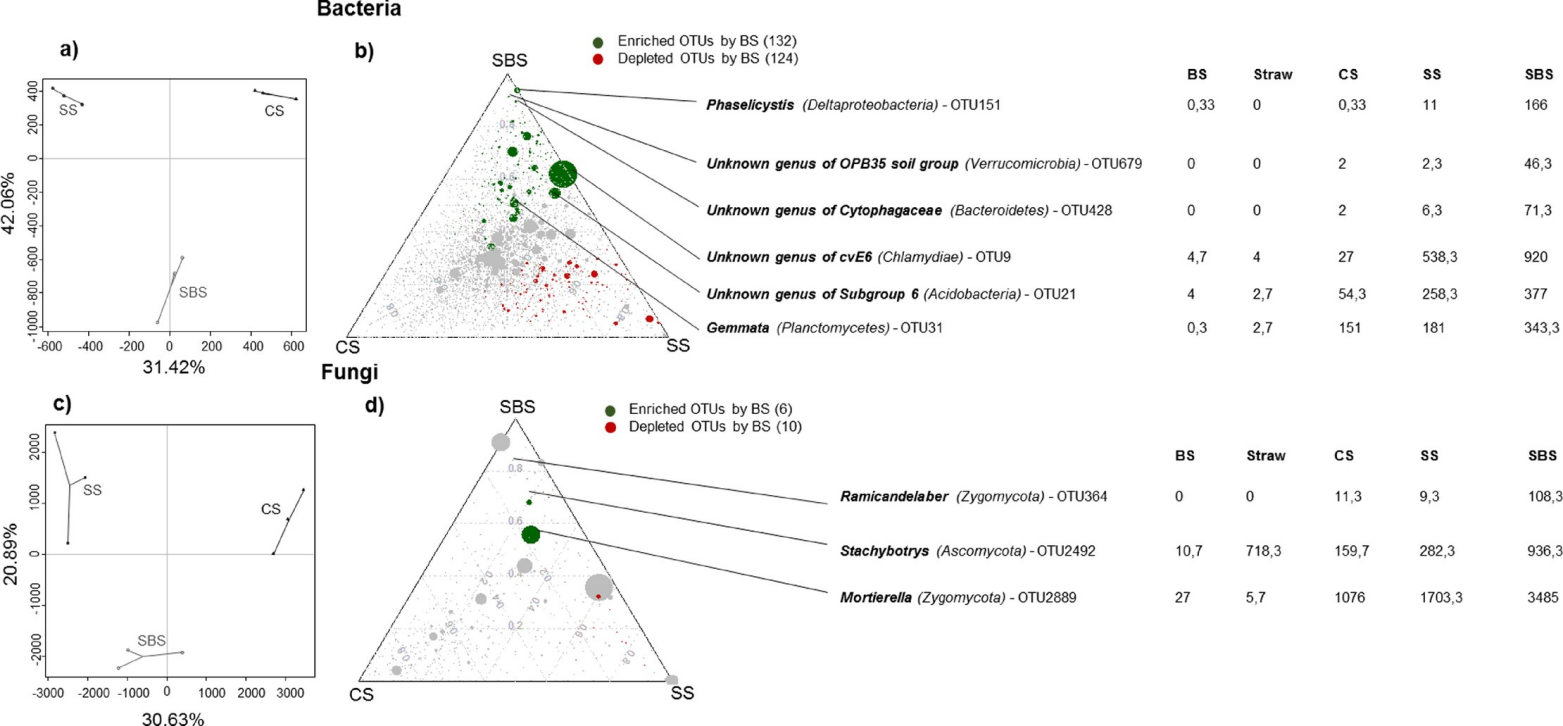
**a and c) Powered partial least squares discriminant analysis (PPLS-DA) describing the bacterial and fungal community structures at the OTU level.** The three soil treatments (CS, SS, SBS) exhibited significant different compositions in terms of their bacterial and fungal communities. The CS groups represent the soil control samples, the SS groups correspond to the soil with straw samples and the SBS groups represent the soil with straw and BS. **b and d) Ternary plots describing the distribution of the bacterial and fungal OTUs between the soil treatments (grey circles) showing the enriched (green circles) and depleted (red circles) OTUs after the BS amendment.** This analysis was performed on the relative abundances for the bacteria and fungi matrices. Each circle depicts one individual OTU. The size of the circle reflects the relative abundance (RA) of the OTU. The position of each circle is determined by the contribution of the indicated compartments to the RA. An extract from S2 and S3 Tables is presented which shows the RA and affiliation of some of the most abundant enriched OTUs.

By analyzing the effect of the BS on the structure of the soil microbial community, significant impacts were observed on both bacteria and fungi but not on archaea, (PPLS-DA analysis, *P* = 0.004 and 73.5% of variance explained, *P* = 0.012 and 51.5% of variance explained and, *P =* 0.65 and 62.1% of variance explained, respectively, (Fig 3A and 3C). The community structures of the soil bacteria and fungi were affected by the addition of both straw and the BS. These results are in agreement with those obtained by Monard et al. [78] and Pascault et al. [50]: the input of fresh OM (carbon substrate or wheat residues, respectively) on the soil induced changes in the soil microbial community structures by selecting and stimulating specific groups of bacteria.

An ANOVA was performed in order to identify the bacterial and fungal OTUs that were responsible for the differences that were observed in the communities when the BS was added (Fig 3B and 3D). Out of a total of 8 697 bacterial OTUs and 842 fungal OTUs, 132 and 6 of these OTUS, respectively, were enriched after the addition of the BS, except for fungal OTU2492 which was also highly detected in the straw (718.3 ± 76.8 sequences). Among the bacterial and fungal enriched OTUs, 83 were specific to the three soil microcosms and the other OTUs were detected at low levels in the BS and/or straw alone. Furthermore, according to the BLASTn analysis of the representative sequences of these enriched OTUs, they were all highly similar to the sequences obtained from the microorganisms extracted from the soil environment. This further supports the idea that the BS acted by stimulating specific indigenous microorganisms in the soil.

Among the most enriched OTUs in the SBS treatment compared to the SS treatment (Fig 3B), specific groups of bacteria were detected such as an unknown genus within the *Cytophagaceae* family (S2 Table; OTU428, OTU495, OTU1016, OTU1771, OTU2157) known to degrade complex carbohydrates [79], *Phaselicystis* sp. (OTU151) which appears to be distributed in soil samples containing decaying plant materials [80], an unknown genus within the *Verrucomicrobia* phylum (OTU679, OTU1362, OTU1883, OTU570, OTU1069, OTU959, OTU3594, OTU1413) known to have an abundance that is positively correlated with recalcitrant C compounds [81] and *Pseudomonas* sp. (OTU1435, OTU942, OTU41, OTU3, OTU596) which are ecological opportunists (r-strategist) [61]. Two fungal OTUs were enriched in the SBS treatment compared to the SS treatment. They were highly similar to *Ramicandelaber* sp. (OTU364) and *Mortierella* sp. (OTU2889) which belong to the phylum *Zygomycota*. *Zygomycota* are known to exhibit a wide range of functional capabilities and to degrade both labile and recalcitrant compounds [76,77]. Moreover, *Ramicandelaber* sp. and *Mortierella* sp. are usually saprophytic in soil [82] and *Mortierella* sp. are P-solubilizing fungi and have been shown to increase urease activity when inoculated in the rhizosphere of *Malvaceae* [83]. This involvement in the P and N cycles in the soil should have a positive effect on plant growth. Most of the enriched OTUs detected were linked to groups that contain known bacterial and fungal decomposers.

## Conclusion

After seven weeks of incubation in soil microcosms, the biostimulant (BS) applied on the straw residues in the soil significantly improved the orgC mineralization, increased the soil microbial biomass and induced changes in both the soil bacterial and fungal communities. At the phylum level, the action of the BS was related to subtle changes in the composition of the soil indigenous bacterial and fungal communities presenting different functional strategies with regards to the use of soil carbon. However, no significant changes in the archaeal community could be identified. At the OTU level, some OTUs were enriched in the presence of the biostimulant and were identified as decomposers able to degrade both labile and more recalcitrant organic substrates suggesting that specific soil bacterial and fungal OTUs were recruited therefore leading to lowest soil microbial richness and diversity. To confirm this possible recruitment by the BS, further analyses using labelled ^13^C straw would be useful to identify the active microbial decomposers and to determine if the straw and BS inputs could have promoted a priming effect. These changes in the soil indigenous diversity induced by the BS might support the activator effect of the BS observed on soil OM mineralization. This cannot be attributed to either (i) the negligible orgC, total N contents of the BS, or (ii) the inoculation of specific microorganisms naturally present in the BS. Thus, the BS may act through other ways. Through its pH neutralizing effect, it may have induced changes in the bacterial and fungal communities, and/or through the supply of amino acids, micronutrients or growth factors that stimulate the microbial communities. Further dedicated analyses are needed to confirm this hypothesis.

## Supporting information

S1 Table Number of remaining sequences and OTUs for bacteria, archaea and fungi after each steps of Frogs and PIPITS pipelines for the 9 soil microcosms and the BS and straw samples (n = 6). (PDF)

S2 Table Relative abundance and taxonomy affiliation of each enriched OTUs, in SBS compared to SS, in BS, straw and soil samples. BS: biostimulants samples, CS: control soil, SS: soil with straw, SBS: soil with straw and BS (ANOVA P<0.05).
(PDF)

S3 Table Relative abundance and taxonomy affiliation of each depleted OTUs, in SBS compared to SS, in BS, straw and soil samples. BS: biostimulants samples, CS: control soil, SS: soil with straw, SBS: soil with straw and BS (ANOVA P<0.05).
(PDF)

S1 Fig Rarefaction curves of bacteria, fungi and archaea in soil, BS and straw samples. BS: biostimulant samples, CS: control soil, SS: soil with straw, SBS: soil with straw and BS.
(PDF)

S2 Fig Rarefaction curves of bacteria, fungi and archaea in soil after normalization: CS: Control soil, SS: Soil with straw, SBS: Soil with straw and BS. (PDF)

S3 Fig Rarefaction curves of bacteria, fungi and archaea in biostimulant and straw after normalization: BS: biostimulant. (PDF)
