# Supplementary material for: Specific recruitment of soil bacteria and fungi decomposers following a biostimulant application increased crop residues mineralization
Source: PLoS One. 2018 Dec 31;13(12):e0209089. doi: 10.1371/journal.pone.0209089 (PMC6312294; doi:10.1371/journal.pone.0209089)
Supplement: S1 Table — (PDF) [file pone.0209089.s001.pdf]

Microbial group	Pipeline	Step	Number of sequences	Number of OTUs
Bacteria	Frogs	initial	1 325 903	-
		pre-process	1 094 023	-
		clustering (swarm, d=3)	1 094 023	79 365
		remove chimera	1 089 641	77 210
		Filters	991 027	8698
	R studio	Normalization (soil microcosms, n=9)	466 461	7755
		Normalization (BS and straw, n=6)	254 838	2283
Archaea	Frogs	initial	1 955 564	-
		pre-process	1 485 630	-
		clustering (swarm, d=3)	1 485 630	35 551
		remove chimera	1 485 534	35 455
		Filters	1 439 039	53
	R studio	Normalization (soil microcosms, n=9)	658 278	50
		Normalization (BS and straw, n=6)	29 052	28
Fungi	PIPITS	initial	2 607 580	-
		pre-process	2 109 448	-
		With the ITS1 region	2 106 149	-
		remove chimera<br>clustering (97% similarity)	2 059 802	3274
		Filters	2 005 465	842
	R studio	Normalization (soil microcosms, n=9)	240 021	698
		Normalization (BS and straw, n=6)	617 034	493
