# Supplementary material for: Specific recruitment of soil bacteria and fungi decomposers following a biostimulant application increased crop residues mineralization
Source: PLoS One. 2018 Dec 31;13(12):e0209089. doi: 10.1371/journal.pone.0209089 (PMC6312294; doi:10.1371/journal.pone.0209089)
Supplement: S2 Table — BS: biostimulants samples, CS: control soil, SS: soil with straw, SBS: soil with straw and BS (ANOVA P<0.05). (PDF) [file pone.0209089.s002.pdf]

Groups	OTU name	Treatments					X times more	Taxonomy					
		BS	Straw	CS	SS	SBS		Phylum	Class	Order	Family	Genus	Species
Bacteria	OTU679	0	0	2 ± 2	2,3 ± 0,6	46,3 ± 8,1	19,9	Verrucomicrobia	OPB35 soil group	unknown order	unknown family	unknown genus	unknown species
	OTU151	0,3 ± 0,6	0	0,3 ± 0,6	11 ± 2,6	166 ± 8	15,1	Proteobacteria	Deltaproteobacteria	Myxococcales	Phaselicystidaceae	Phaselicystis	unknown species
	OTU428	0	0	2 ± 2,6	6,3 ± 4,5	71,3 ± 42,1	11,3	Bacteroidetes	Cytophagia	Cytophagales	Cytophagaceae	unknown genus	unknown species
	OTU495	0	0	10 ± 7	5,7 ± 1,1	56,3 ± 20,7	9,9	Bacteroidetes	Cytophagia	Cytophagales	Cytophagaceae	unknown genus	unknown species
	OTU1435	17,7 ± 7,6	21,3 ± 16,9	10,3 ± 7,6	3,3 ± 3,2	31,3 ± 11	9,4	Proteobacteria	Gammaproteobacteria	Pseudomonadales	Pseudomonadaceae	Pseudomonas	unknown species
	OTU1620	0	0	1,7 ± 2,1	2 ± 1	16,3 ± 2,1	8,2	Planctomycetes	OM190	unknown order	unknown family	unknown genus	unknown species
	OTU1991	0	0	2,3 ± 2,3	1,3 ± 0,6	10,7 ± 2,5	8	Acidobacteria	Subgroup 6	unknown order	unknown family	unknown genus	unknown species
	OTU588	0,3 ± 0,6	0	4,7 ± 2,9	6 ± 3	47,7 ± 10,3	7,9	Firmicutes	Bacilli	Bacillales	unknown family	unknown genus	unknown species
	OTU288	0	0	8 ± 4	11,7 ± 1,5	90 ± 6,1	7,7	TM6 (Dependentiae)	unknown class	unknown order	unknown family	unknown genus	unknown species
	OTU1362	0	0	4,7 ± 0,6	2,3 ± 1,1	17,7 ± 2,1	7,6	Verrucomicrobia	OPB35 soil group	unknown order	unknown family	unknown genus	unknown species
	OTU2114	0	0	0,7 ± 0,6	3 ± 3,5	21,7 ± 12	7,2	Planctomycetes	Planctomycetacia	Planctomycetales	Planctomycetaceae	Planctomyces	unknown species
	OTU1883	0	0	0	2,7 ± 1,5	19 ± 8,7	7,1	Verrucomicrobia	Spartobacteria	Chthoniobacterales	DA101 soil group	unknown genus	unknown species
	OTU1303	0	0	4,3 ± 0,6	3,3 ± 1,5	23,7 ± 9,3	7,1	Proteobacteria	Betaproteobacteria	Nitrosomonadales	Nitrosomonadaceae	unknown genus	unknown species
	OTU223	0	0	22 ± 9,2	15 ± 2,6	96,3 ± 11,9	6,4	Gemmatimonadetes	Gemmatimonadetes	Gemmatimonadales	Gemmatimonadaceae	unknown genus	unknown species
	OTU2356	0	0	1,7 ± 1,5	2 ± 1	12,7 ± 4,5	6,3	Planctomycetes	Planctomycetacia	Planctomycetales	Planctomycetaceae	unknown genus	unknown species
	OTU1016	0	0	14,7 ± 16	7,7 ± 4,9	47,3 ± 13,5	6,2	Bacteroidetes	Cytophagia	Cytophagales	Cytophagaceae	unknown genus	unknown species
	OTU1259	0,3 ± 0,6	0,7 ± 1,1	1,3 ± 1,2	3,3 ± 0,6	20 ± 7,9	6	Actinobacteria	Actinobacteria	Streptosporangiales	Streptosporangiaceae	Nonomuraea	unknown species
	OTU1127	0	0,3 ± 0,6	1,3 ± 1,5	5 ± 1	27,7 ± 6,4	5,5	BRC1	unknown class	unknown order	unknown family	unknown genus	unknown species
	OTU956	0	0	5 ± 1,7	8 ± 1	42 ± 3	5,3	Acidobacteria	Blastocatellia	Blastocatellales	Blastocatellaceae (Subgroup 4)	unknown genus	unknown species
	OTU319	0,7 ± 1,1	0	7,3 ± 8,5	17 ± 3	84 ± 5,3	4,9	Planctomycetes	Phycisphaerae	Tepidisphaerales	Tepidisphaeraceae	unknown genus	unknown species
	OTU942	0	0	8,3 ± 1,5	5,7 ± 3,2	27,7 ± 1,5	4,9	Proteobacteria	Gammaproteobacteria	Pseudomonadales	Pseudomonadaceae	Pseudomonas	unknown species
	OTU1484	0	0	0	3,7 ± 2,3	17,7 ± 3,2	4,8	Planctomycetes	OM190	unknown order	unknown family	unknown genus	unknown species
	OTU629	0	0	13,7 ± 4,6	8 ± 5,3	37 ± 18,73	4,6	Bacteroidetes	Sphingobacteriia	Sphingobacteriales	Chitinophagaceae	Terrimonas	unknown species
	OTU848	0	0	6 ± 1,7	6,7 ± 6,4	30,3 ± 6,5	4,6	Proteobacteria	Betaproteobacteria	TRA3-20	unknown family	unknown genus	unknown species
	OTU326	0	0,3 ± 0,6	26,7 ± 3,8	13 ± 3,5	57,7 ± 12,4	4,4	Acidobacteria	Subgroup 6	unknown order	unknown family	unknown genus	unknown species
	OTU41	19 ± 14,1	10 ± 7,5	69,3 ± 9	86,7 ± 24,7	369,3 ± 80,2	4,3	Proteobacteria	Gammaproteobacteria	Pseudomonadales	Pseudomonadaceae	Pseudomonas	unknown species
	OTU597	0	0	3,7 ± 1,5	10,3 ± 5,5	44 ± 16,1	4,3	Planctomycetes	Planctomycetacia	Planctomycetales	Planctomycetaceae	unknown genus	unknown species
	OTU1321	0	0	15 ± 2	7 ± 1,7	29,6 ± 4,2	4,2	Acidobacteria	Subgroup 6	unknown order	unknown family	unknown genus	unknown species
	OTU58	0	0,7 ± 0,6	21,7 ± 6,7	67,7 ± 12,4	286,7 ± 59,9	4,2	Planctomycetes	Planctomycetacia	Planctomycetales	Planctomycetaceae	Pirellula	unknown species
	OTU519	0,7 ± 1,1	0	4 ± 1	12 ± 1,7	50 ± 16,8	4,2	Proteobacteria	Gammaproteobacteria	HTA4	unknown family	unknown genus	unknown species
	OTU1660	0	0	2,3 ± 3,2	3,3 ± 1,5	13,7 ± 3	4,1	Acidobacteria	Blastocatellia	Blastocatellales	Blastocatellaceae (Subgroup 4)	RB41	unknown species
	OTU610	0	0,3 ± 0,6	17 ± 7,8	10 ± 6,2	40,3 ± 12,4	4	Acidobacteria	Subgroup 6	unknown order	unknown family	unknown genus	unknown species
	OTU1771	0	0	4 ± 1	2,7 ± 1,1	10,7 ± 3,5	4	Bacteroidetes	Cytophagia	Cytophagales	Cytophagaceae	unknown genus	unknown species
	OTU1051	0,3 ± 0,6	0	10 ± 3,6	5,7 ± 2,5	22 ± 1	3,9	Bacteroidetes	Sphingobacteriia	Sphingobacteriales	Chitinophagaceae	Ferruginibacter	unknown species
	OTU3139	0	0	0,3 ± 0,6	1,7 ± 1,1	6,3 ± 2,3	3,8	Proteobacteria	Deltaproteobacteria	Myxococcales	Blrii41	unknown genus	unknown species
	OTU971	0	0	23 ± 2,6	7,3 ± 2,5	27,7 ± 4,9	3,8	Bacteroidetes	Sphingobacteriia	Sphingobacteriales	Chitinophagaceae	unknown genus	unknown species
	OTU251	0	0	2,7 ± 2,5	25 ± 11,8	90,7 ± 30,3	3,6	Proteobacteria	Gammaproteobacteria	Legionellales	Coxiellaceae	Aquicella	unknown species
	OTU843	0	0	0,3 ± 0,6	14 ± 7	50,7 ± 22,5	3,6	Chloroflexi	Anaerolineae	Anaerolineales	Anaerolineaceae	unknown genus	unknown species
	OTU847	0	0,3 ± 0,6	11,3 ± 5,6	7 ± 4,4	25,3 ± 7,2	3,6	Acidobacteria	Subgroup 6	unknown order	unknown family	unknown genus	unknown species
	OTU732	0	0	1 ± 1,7	11 ± 2	39,3 ± 8,7	3,6	Planctomycetes	Planctomycetacia	Planctomycetales	Planctomycetaceae	Planctomyces	unknown species
	OTU1538	0	0	0	5,3 ± 3,5	18,7 ± 3	3,5	Spirochaetae	Spirochaetes	Spirochaetales	Leptospiraceae	Turneriella	unknown species
	OTU2673	0	0	4,3 ± 1,5	1,3 ± 0,6	4,7 ± 1,5	3,5	Chlamydiae	Chlamydiae	Chlamydiales	Parachlamydiaceae	unknown genus	Neochlamydia sp.
	OTU3116	0	0	2,7 ± 1,1	2 ± 1	7 ± 2,6	3,5	Chloroflexi	SBR2076	unknown order	unknown family	unknown genus	unknown species
	OTU206	0,3 ± 0,6	0	41,3 ± 6,5	27,3 ± 4,5	95 ± 10,4	3,5	Planctomycetes	Planctomycetacia	Planctomycetales	Planctomycetaceae	Pirellula	unknown species
	OTU570	0	0	3 ± 1	18,3 ± 14,6	62 ± 10	3,4	Verrucomicrobia	OPB35 soil group	unknown order	unknown family	unknown genus	unknown species
	OTU1285	0	0	0	6,3 ± 5,8	21,3 ± 6,5	3,4	Proteobacteria	Deltaproteobacteria	Myxococcales	Haliangiaceae	Haliangium	unknown species
	OTU3	4318,7 ± 4965,1	1525,3 ± 241,4	4,3 ± 2,1	6,7 ± 2,9	22,3 ± 4,7	3,4	Proteobacteria	Gammaproteobacteria	Pseudomonadales	Pseudomonadaceae	Pseudomonas	unknown species
	OTU2859	0	0	0,7 ± 1,1	2 ± 1	6,7 ± 2,1	3,3	Planctomycetes	Planctomycetacia	Planctomycetales	Planctomycetaceae	Gemmata	unknown species
	OTU6532	0	0	3,7 ± 0,6	1 ± 0	3,3 ± 11,1	3,3	Proteobacteria	Gammaproteobacteria	Legionellales	Coxiellaceae	Aquicella	unknown species
	OTU762	0	0	2 ± 1,7	10,7 ± 8,9	35,3 ± 11,4	3,3	Proteobacteria	Deltaproteobacteria	Myxococcales	Haliangiaceae	Haliangium	unknown species
	OTU448	0	0,3 ± 0,6	3 ± 3	15,3 ± 5,5	50,7 ± 9,7	3,3	Planctomycetes	Planctomycetacia	Planctomycetales	Planctomycetaceae	Pirellula	unknown species
	OTU2713	0	0,3 ± 0,6	2 ± 1	3,7 ± 1,1	12 ± 2,6	3,3	Proteobacteria	Betaproteobacteria	Nitrosomonadales	Nitrosomonadaceae	unknown genus	unknown species
	OTU278	0	0	10 ± 1,7	27,3 ± 5,5	88,7 ± 33,3	3,2	Gemmatimonadetes	Gemmatimonadetes	Gemmatimonadales	Gemmatimonadaceae	unknown genus	unknown species
	OTU316	0	0,3 ± 0,6	15 ± 5	22,3 ± 6,4	71,3 ± 19,7	3,2	Planctomycetes	Planctomycetacia	Planctomycetales	Planctomycetaceae	Pir4 lineage	unknown species
	OTU2723	0	0	7,7 ± 2,5	3,7 ± 2,9	11,7 ± 3	3,2	Proteobacteria	Alphaproteobacteria	Rickettsiales	Holosporaceae	unknown genus	unknown species
	OTU1069	0	0	8,3 ± 2,3	7,7 ± 6,7	24,3 ± 7	3,2	Verrucomicrobia	Spartobacteria	Chthoniobacterales	Chthoniobacteraceae	Chthoniobacter	unknown species
	OTU70	3	0	79 ± 43,5	63,7 ± 21,2	201,7 ± 29,1	3,2	Planctomycetes	Planctomycetacia	Planctomycetales	Planctomycetaceae	Gemmata	unknown species
	OTU521	0	0,3 ± 0,6	20 ± 8,7	14 ± 3,5	44,3 ± 8,6	3,2	Planctomycetes	Phycisphaerae	Tepidisphaerales	Tepidisphaeraceae	unknown genus	unknown species
	OTU2188	0	0	4,7 ± 2,1	2 ± 1	6,3 ± 1,5	3,2	Gemmatimonadetes	Gemmatimonadetes	Gemmatimonadales	Gemmatimonadaceae	unknown genus	unknown species
	OTU580	0	0,3 ± 0,6	2 ± 2	14,7 ± 4,9	45,7 ± 8,7	3,1	Chlamydiae	Chlamydiae	Chlamydiales	Parachlamydiaceae	Neochlamydia	unknown species
	OTU2173	0	0	6,7 ± 4,7	4,7 ± 3	14,3 ± 3,2	3,1	Chlorobi	Chlorobia	Chlorobiales	SJA-28	unknown genus	unknown species
	OTU1531	1 ± 1	0	2,7 ± 2,1	7 ± 4,6	21,3 ± 4,5	3,1	Actinobacteria	Actinobacteria	Streptomycetales	Streptomycetaceae	Streptomyces	unknown species
	OTU572	0	1 ± 1,7	27,3 ± 9,3	12,3 ± 5,5	37,3 ± 7,2	3	Gemmatimonadetes	Gemmatimonadetes	Gemmatimonadales	Gemmatimonadaceae	unknown genus	unknown species
	OTU959	0	0	2,7 ± 2,5	9 ± 4,6	27 ± 1	3	Verrucomicrobia	Spartobacteria	Chthoniobacterales	Chthoniobacteraceae	Chthoniobacter	unknown species
	OTU3594	0	0	0	2 ± 1	6 ± 1	3	Verrucomicrobia	Spartobacteria	Chthoniobacterales	Chthoniobacteraceae	Chthoniobacter	unknown species
	OTU642	0	0	33,3 ± 1,5	5 ± 2,6	14,7 ± 5,5	2,9	Acidobacteria	Blastocatellia	Blastocatellales	Blastocatellaceae (Subgroup 4)	RB41	unknown species
	OTU1447	0	0	0	7 ± 5,2	20,3 ± 4	2,9	Chloroflexi	Anaerolineae	Anaerolineales	Anaerolineaceae	unknown genus	unknown species
	OTU270	1 ± 1,7	0,3 ± 0,6	38,3 ± 9,3	20,7 ± 2,5	59,3 ± 9	2,9	Gemmatimonadetes	Gemmatimonadetes	Gemmatimonadales	Gemmatimonadaceae	unknown genus	unknown species
	OTU675	0	0	28,7 ± 7,8	9,7 ± 2,9	27,7 ± 5,8	2,9	Proteobacteria	Betaproteobacteria	Nitrosomonadales	Nitrosomonadaceae	Nitrosospira	unknown species
	OTU1263	0	0	17,3 ± 1,1	4,7 ± 3,2	13,3 ± 3,8	2,9	Bacteroidetes	Sphingobacteriia	Sphingobacteriales	Saprospiraceae	unknown genus	unknown species
	OTU726	0	0	8,7 ± 2,5	10,3 ± 5,5	29,3 ± 8	2,8	Proteobacteria	Betaproteobacteria	TRA3-20	unknown family	unknown genus	unknown species
	OTU536	0	0	28,7 ± 6,7	8,7 ± 2,3	24 ± 1,7	2,8	Acidobacteria	Subgroup 5	unknown order	unknown family	unknown genus	unknown species
	OTU2911	0	0	0,7 ± 1,1	2,7 ± 1,1	7,3 ± 2,5	2,8	Acidobacteria	Blastocatellia	Blastocatellales	Blastocatellaceae (Subg	DS-100	unknown species
	OTU637	0,3 ± 0,6	0,3 ± 0,6	4,3 ± 5,8	11,7 ± 2,3	32 ± 7,5	2,7	Planctomycetes	Planctomycetacia	Planctomycetales	Planctomycetaceae	Pirellula	unknown species
	OTU67	36 ± 20,9	37 ± 12,9	56,7 ± 13,6	48,3 ± 11,1	132,3 ± 42,6	2,7	Actinobacteria	Actinobacteria	Micrococcales	Micrococcaceae	unknown genus	unknown species
	OTU1159	0	0,3 ± 0,6	5,7 ± 1,5	6 ± 1,7	16,3 ± 3,5	2,7	Acidobacteria	Blastocatellia	Blastocatellales	Blastocatellaceae (Subgroup 4)	DS-100	unknown species
	OTU329	0	0	23,7 ± 13,6	31 ± 12,5	82,7 ± 10,7	2,7	Bacteroidetes	Sphingobacteriia	Sphingobacteriales	Saprospiraceae	unknown genus	unknown species
	OTU390	0	0	12,7 ± 5,5	22 ± 8,2	58,3 ± 16,6	2,7	Planctomycetes	Planctomycetacia	Planctomycetales	Planctomycetaceae	Planctomyces	unknown species
	OTU696	1,3 ± 1,1	0	2,7 ± 1,5	13 ± 9	34 ± 1	2,6	Planctomycetes	Planctomycetacia	Planctomycetales	Planctomycetaceae	Planctomyces	unknown species
	OTU1994	0	0	1 ± 1	5 ± 2,6	13 ± 3	2,6	Planctomycetes	Planctomycetacia	Planctomycetales	Planctomycetaceae	Planctomyces	unknown species
	OTU235	0	1 ± 1,7	14,3 ± 13,6	32 ± 7,9	83 ± 9,5	2,6	Planctomycetes	Planctomycetacia	Planctomycetales	Planctomycetaceae	Pirellula	unknown species
	OTU371	0	0	19 ± 9	19,7 ± 7,7	51 ± 16,6	2,6	Bacteroidetes	Sphingobacteriia	Sphingobacteriales	Saprospiraceae	unknown genus	unknown species
	OTU862	1,3 ± 2,3	0,3 ± 0,6	20,3 ± 7	9 ± 6,1	23,3 ± 3	2,6	Planctomycetes	Phycisphaerae	Tepidisphaerales	Tepidisphaeraceae	unknown genus	unknown species
	OTU186	4,3 ± 6,7	0,7 ± 1,1	60,3 ± 11,1	64 ± 3	165,7 ± 20,3	2,6	Acidobacteria	Solibacteres	Solibacterales	Solibacteraceae (Subgroup 3)	Bryobacter	unknown species
	OTU1109	0	0	1,3 ± 2,3	11 ± 567	28,3 ± 9	2,6	Bacteroidetes	Sphingobacteriia	Sphingobacteriales	Chitinophagaceae	Chitinophaga	bacterium enrichment culture
	OTU485	0	0	18 ± 7,9	15,3 ± 5,9	39,3 ± 4,7	2,6	Acidobacteria	Subgroup 6	unknown order	unknown family	unknown genus	unknown species
	OTU1480	0	0	0,7 ± 1,1	7,7 ± 3,1	19,7 ± 3,2	2,6	Actinobacteria	Actinobacteria	Propionibacteriales	Nocardioidaceae	Kribbella	unknown species
	OTU2157	0	0	2,3 ± 1,1	5,3 ± 0,6	13,7 ± 5,5	2,6	Bacteroidetes	Cytophagia	Cytophagales	Cytophagaceae	Ohtaekwangia	unknown species
	OTU274	0	2 ± 2,6	45 ± 15,4	35,3 ± 10	89,3 ± 7,5	2,5	Gemmatimonadetes	Gemmatimonadetes	Gemmatimonadales	Gemmatimonadaceae	unknown genus	unknown species
	OTU1178	0	0	0	7,7 ± 0,6	19,3 ± 5	2,5	Proteobacteria	Gammaproteobacteria	Xanthomonadales	Xanthomonadaceae	Lysobacter	Lysobacter sp.
	OTU61	0	0,7 ± 0,6	32 ± 15	90 ± 20,4	219,7 ± 37,6	2,4	Planctomycetes	Planctomycetacia	Planctomycetales	Planctomycetaceae	Planctomyces	Planctomyces sp.
	OTU1413	0	0,3 ± 0,6	0,3 ± 0,6	8,3 ± 3	20,3 ± 1,5	2,4	Verrucomicrobia	Spartobacteria	Chthoniobacterales	Chthoniobacterales Incertae Sedis	Terrimicrobium	unknown species
Bacteria	OTU1242	0	0	7,7 ± 3,5	6,3 ± 0,6	15,3 ± 5	2,4	Acidobacteria	Subgroup 6	unknown order	unknown family	unknown genus	unknown species
	OTU2123	0	0	0	4,3 ± 1,5	10,3 ± 3,2	2,4	Proteobacteria	Gammaproteobacteria	Xanthomonadales	unknown family	unknown genus	unknown species
	OTU3413	0	0	0,7 ± 0,6	4,3 ± 2,5	10,3 ± 3,2	2,4	Proteobacteria	Deltaproteobacteria	Myxococcales	Nannocystaceae	Nannocystis	unknown species
	OTU1579	0	0	0,7 ± 1,1	8,3 ± 1,1	19,3 ± 6,5	2,3	Proteobacteria	Deltaproteobacteria	Bdellovibrionales	Bdellovibrionaceae	OM27 clade	unknown species
	OTU1245	0	0	7 ± 3,5	5,3 ± 0,6	12,3 ± 2,31	2,3	Acidobacteria	Subgroup 25	unknown order	unknown family	unknown genus	unknown species
	OTU618	0	0	16 ± 7,2	16,3 ± 7,4	37,7 ± 2,5	2,3	Planctomycetes	Planctomycetacia	Planctomycetales	Planctomycetaceae	unknown genus	unknown species
	OTU502	0	0	17,7 ± 4,6	15 ± 5,6	34,3 ± 6,8	2,3	Acidobacteria	Subgroup 6	unknown order	unknown family	unknown genus	unknown species
	OTU1211	0	0	23,3 ± 2,3	10,7 ± 5,7	24,3 ± 5,5	2,3	Acidobacteria	Subgroup 6	unknown order	unknown family	unknown genus	unknown species
	OTU957	0,3 ± 0,6	0	3,7 ± 3,2	10,7 ± 4,5	24,3 ± 6,3	2,3	Proteobacteria	Betaproteobacteria	Burkholderiales	Comamonadaceae	unknown genus	unknown species
	OTU585	0	0	14,7 ± 8,1	14,3 ± 4,2	32,3 ± 7,8	2,3	Gemmatimonadetes	Gemmatimonadetes	Gemmatimonadales	Gemmatimonadaceae	Gemmatimonas	unknown species
	OTU596	0	0	16 ± 4,56	14 ± 6,1	31,3 ± 5,5	2,2	Proteobacteria	Gammaproteobacteria	Pseudomonadales	Pseudomonadaceae	Pseudomonas	unknown species
	OTU598	0	0	6 ± 6,1	17,7 ± 9,3	39,3 ± 7,8	2,2	Bacteroidetes	Sphingobacteriia	Sphingobacteriales	Chitinophagaceae	Terrimonas	unknown species
	OTU524	0	0	17,3 ± 6,5	15 ± 2	33,3 ± 10	2,2	Planctomycetes	Planctomycetacia	Planctomycetales	Planctomycetaceae	Pir4 lineage	unknown species
	OTU100	0,67 ± 1,15	0,7 ± 1,1	109,7 ± 10,1	64 ± 9,5	137,7 ± 38,5	2,2	Planctomycetes	Planctomycetacia	Planctomycetales	Planctomycetaceae	Pirellula	unknown species
	OTU920	0,3 ± 0,6	0	7,3 ± 2,3	8,7 ± 4	18,3 ± 4,7	2,1	Firmicutes	Bacilli	Bacillales	unknown family	unknown genus	unknown species
	OTU872	0	0	11 ± 3,6	12 ± 5,3	25 ± 6	2,1	Planctomycetes	Planctomycetacia	Planctomycetales	Planctomycetaceae	Schlesneria	unknown species
	OTU219	0,3 ± 0,6	0	44,3 ± 5,5	52 ± 9,6	107 ± 14	2,1	Proteobacteria	Betaproteobacteria	TRA3-20	unknown family	unknown genus	unknown species
	OTU1041	0	0	2,7 ± 1,5	9,3 ± 2,5	19 ± 5,6	2	Planctomycetes	Phycisphaerae	mle1-8	unknown family	unknown genus	unknown species
	OTU508	0	0	44 ± 11,5	18,3 ± 3,5	37 ± 3,6	2	Acidobacteria	Blastocatellia	Blastocatellales	Blastocatellaceae (Subgroup 4)	unknown genus	unknown species
	OTU340	0	0,7 ± 1,1	9 ± 4	28,3 ± 6,1	56,3 ± 10,2	2	Planctomycetes	Planctomycetacia	Planctomycetales	Planctomycetaceae	Planctomyces	unknown species
	OTU442	0	0	26,3 ± 3,8	18,7 ± 2,9	37 ± 7,2	2	Gemmatimonadetes	Gemmatimonadetes	Gemmatimonadales	Gemmatimonadaceae	unknown genus	unknown species
	OTU210	0	0,7 ± 1,1	35,3 ± 9,5	33,3 ± 10	64 ± 4,6	1,9	Proteobacteria	Betaproteobacteria	Nitrosomonadales	Nitrosomonadaceae	unknown genus	unknown species
	OTU167	0	0	22,7 ± 7,2	48,7 ± 10,7	93 ± 11,5	1,9	Planctomycetes	Planctomycetacia	Planctomycetales	Planctomycetaceae	Gemmata	unknown species
	OTU31	0,3 ± 0,6	2,7 ± 4,6	151 ± 69,6	181 ± 36,1	343,3 ± 46,6	1,9	Planctomycetes	Planctomycetacia	Planctomycetales	Planctomycetaceae	Gemmata	unknown species
	OTU82	0,67 ± 1,15	0	50,7 ± 19,6	93,7 ± 30,3	177,7 ± 45	1,9	Proteobacteria	Gammaproteobacteria	Xanthomonadales	unknown family	unknown genus	unknown species
	OTU464	0	0	8 ± 2,6	21,3 ± 6,5	39,3 ± 8,1	1,8	Bacteroidetes	Sphingobacteriia	Sphingobacteriales	Chitinophagaceae	Flavisolibacter	unknown species
	OTU551	0	0	12,7 ± 3,5	27,7 ± 4,2	48,7 ± 7,4	1,8	Planctomycetes	Planctomycetacia	Planctomycetales	Planctomycetaceae	unknown genus	unknown species
	OTU895	0	0	11,3 ± 4,7	15 ± 4	26,3 ± 3,5	1,8	Proteobacteria	Alphaproteobacteria	Rhizobiales	MNG7	unknown genus	unknown species
	OTU1764	0	0	1 ± 1	5,3 ± 1,5	9,3 ± 0,6	1,8	Planctomycetes	OM190	unknown order	unknown family	unknown genus	unknown species
	OTU120	0	0	60,3 ± 13,3	76 ± 6,6	130 ± 14	1,7	Planctomycetes	Planctomycetacia	Planctomycetales	Planctomycetaceae	unknown genus	unknown species
	OTU9	4,67 ± 4,72	4 ± 5,2	27 ± 19,9	538,3 ± 31,2	920 ± 229,6	1,7	Chlamydiae	Chlamydiae	Chlamydiales	cvE6	unknown genus	unknown species
	OTU678	0,3 ± 0,6	0	23 ± 2,6	12 ± 4,6	20,3 ± 2,1	1,7	Acidobacteria	Blastocatellia	Blastocatellales	Blastocatellaceae (Subgroup 4)	RB41	unknown species
	OTU199	0,3 ± 0,6	0	27,7 ± 9,3	43,7 ± 12,6	73,3 ± 11,9	1,7	Acidobacteria	Subgroup 6	unknown order	unknown family	unknown genus	unknown species
	OTU460	0,67 ± 1,15	0	18,3 ± 6,7	25,7 ± 1,5	41,7 ± 7,1	1,6	Proteobacteria	Betaproteobacteria	Nitrosomonadales	Nitrosomonadaceae	unknown genus	unknown species
	OTU84	2 ± 3,46	0	77,3 ± 15,3	98,7 ± 16,5	158 ± 11,3	1,6	Bacteroidetes	Sphingobacteriia	Sphingobacteriales	Chitinophagaceae	unknown genus	unknown species
	OTU49	0	1,7 ± 0,6	161,3 ± 20,5	183,7 ± 44,4	285 ± 34,6	1,6	Acidobacteria	Subgroup 6	unknown order	unknown family	unknown genus	unknown species
	OTU277	0,3 ± 0,6	0,3 ± 0,6	46,7 ± 9,5	29,7 ± 2,5	45,7 ± 2,1	1,5	Acidobacteria	Subgroup 6	unknown order	unknown family	unknown genus	unknown species
	OTU21	4 ± 2	2,7 ± 3,8	54,3 ± 23,5	258,3 ± 21,1	377 ± 18,3	1,5	Acidobacteria	Subgroup 6	unknown order	unknown family	unknown genus	unknown species
	OTU718	0	0	10,3 ± 0,59	14,3 ± 3,8	20,3 ± 1,5	1,4	Bacteroidetes	Sphingobacteriia	Sphingobacteriales	Chitinophagaceae	unknown genus	unknown species
	OTU30	2 ± 2,65	4 ± 6,1	235,7 ± 11,7	171,3 ± 5,8	214 ± 14,1	1,3	Proteobacteria	Deltaproteobacteria	Desulfurellales	Desulfurellaceae	H16	unknown species
Fungi	OTU364	0	0	11,3 ± 6	9,3 ± 7,6	108,3 ± 56,4	11,6	Zygomycota	Kickxellomycotina	Kickxellales	Kickxellaceae	Ramicandelaber	Ramicandelaber sp HMH_2010a
	OTU2492	10,7 ± 10,3	718,3 ± 76,8	159,7 ± 12,6	282,3 ± 33,2	936,3 ± 148,2	3,3	Ascomycota	Sordariomycetes	Hypocreales	Hypocreales_Incertae sedis	Stachybotrys	Stachybotrys chartarum
	OTU1898	0	0	0,7 ± 1,1	3 ± 3,5	9,3 ± 2,1	3,1	Unknown	Unknown	Unknown	Unknown	Unknown	Unknown
	OTU3135	1,3 ± 1,5	0	19,7 ± 32,3	111 ± 29,5	303,7 ± 79,1	2,7	Unknown	Unknown	Unknown	Unknown	Unknown	Unknown
	OTU958	0	0	0	3,3 ± 0,6	7,6 ± 2,1	2,3	Unknown	Unknown	Unknown	Unknown	Unknown	Unknown
	OTU2889	27 ± 17,6	5,7 ± 1,1	1076 ± 185,3	1703,3 ± 534	3485 ± 698,8	2	Zygomycota	Mucoromycotina_Incer	Mortierellales	Mortierellaceae	Mortierella	Mortierella elongata
