# Supplementary material for: Specific recruitment of soil bacteria and fungi decomposers following a biostimulant application increased crop residues mineralization
Source: PLoS One. 2018 Dec 31;13(12):e0209089. doi: 10.1371/journal.pone.0209089 (PMC6312294; doi:10.1371/journal.pone.0209089)
Supplement: S3 Table — BS: biostimulants samples, CS: control soil, SS: soil with straw, SBS: soil with straw and BS (ANOVA P<0.05). (PDF) [file pone.0209089.s003.pdf]

| Groups | OTU name | Treatments | | | | | X times less | Taxonomy | | | | | |
|---|---|---|---|---|---|---|---|---|---|---|---|---|---|
| | | BS | Straw | CS | SS | SBS | | Phylum | Class | Order | Family | Genus | Species |
| Bacteria | OTU169 | 0,3 ± 0,6 | 0,3 ± 0,6 | 58 ± 10,1 | 80,7 ± 5,5 | 54 ±11,5 | 6,7 | Verrucomicrobia | OPB35 soil group | unknown order | unknown family | unknown genus | unknown species |
| | OTU553 | 0 | 1,7 ± 2,1 | 8,7 ± 3,8 | 41 ± 8,2 | 25,7 ± 3,2 | 6,3 | Proteobacteria | Gammaproteobacteria | Xanthomonadales | Xanthomonadales Incertae Sedis | Acidibacter | unknown species |
| | OTU279 | 0,7 ± 1,1 | 0 | 43,3 ± 4,2 | 47 ± 5,3 | 29,3 ± 3,5 | 6,2 | Acidobacteria | Subgroup 5 | unknown order | unknown family | unknown genus | unknown species |
| | OTU310 | 0 | 0 | 49,7 ± 6,7 | 63 ± 5 | 38,3 ± 8,9 | 6,1 | Planctomycetes | Phycisphaerae | Tepidisphaerales | Tepidisphaeraceae | unknown genus | unknown species |
| | OTU281 | 0 | 0 | 32 ± 5,2 | 50,7 ± 8,9 | 30,3 ± 5,9 | 6 | Proteobacteria | Betaproteobacteria | Nitrosomonadales | Nitrosomonadaceae | unknown genus | unknown species |
| | OTU1213 | 1,3 ± 2,3 | 0 | 11,7 ± 2,5 | 20,7 ± 3,2 | 12,3 ± 4 | 6 | Proteobacteria | Betaproteobacteria | Rhodocyclales | Rhodocyclaceae | unknown genus | unknown species |
| | OTU57 | 0 | 1 ± 1 | 90,7 ± 4,7 | 166,7 ± 35,5 | 92,7 ± 24,9 | 5,6 | Acidobacteria | Subgroup 6 | unknown order | unknown family | unknown genus | unknown species |
| | OTU683 | 0 | 0,7 ± 1,1 | 23 ± 6,1 | 40,3 ± 2,1 | 22,3 ± 4 | 5,5 | Proteobacteria | Deltaproteobacteria | Myxococcales | Haliangiaceae | Haliangium | unknown species |
| | OTU349 | 0 | 0 | 29,7 ± 5,9 | 38,3 ± 4 | 21 ± 4,4 | 5,5 | Acidobacteria | Subgroup 6 | unknown order | unknown family | unknown genus | unknown species |
| | OTU5161 | 0 | 0 | 0,3 ± 0,6 | 3,7 ± 0,6 | 2 ± 0 | 5,5 | Chloroflexi | Anaerolineae | Anaerolineales | Anaerolineaceae | unknown genus | unknown species |
| | OTU1937 | 0 | 0,3 ± 0,6 | 4 ± 3,6 | 16 ± 2,6 | 8,7 ± 0,6 | 5,4 | Proteobacteria | Betaproteobacteria | TRA3-20 | unknown family | unknown genus | unknown species |
| | OTU176 | 0,3 ± 0,6 | 1,3 ± 2,3 | 65 ± 8,2 | 59,3 ± 6,7 | 31,3 ± 8,6 | 5,3 | Acidobacteria | Acidobacteria | Acidobacteriales | Acidobacteriaceae (Subgroup 1) | unknown genus | unknown species |
| | OTU1108 | 0 | 0 | 37,7 ± 4,9 | 26,7 ± 3,5 | 14 ±1,7 | 5,3 | Bacteroidetes | Sphingobacteriia | Sphingobacteriales | Chitinophagaceae | unknown genus | unknown species |
| | OTU253 | 0,3 ± 0,6 | 1,3 ± 2,3 | 43,7 ± 6,7 | 79 ± 11,1 | 41,3 ± 6,1 | 5,2 | Proteobacteria | Gammaproteobacteria | Xanthomonadales | Xanthomonadaceae | Dokdonella | unknown species |
| | OTU1539 | 0 | 0,3 ± 0,6 | 0,7 ± 1,1 | 16,7 ± 1,5 | 8,7 ± 4,9 | 5,2 | Proteobacteria | Deltaproteobacteria | Myxococcales | Polyangiaceae | Sorangium | unknown species |
| | OTU85 | 0,3 ± 0,6 | 1,7 ± 2,9 | 43,7 ± 14,1 | 147,3 ± 19,5 | 76,3 ± 8,1 | 5,2 | Acidobacteria | Subgroup 5 | unknown order | unknown family | unknown genus | unknown species |
| | OTU308 | 1 ± 1,7 | 0 | 31,7 ± 3,8 | 53 ± 14 | 27,3 ± 9,6 | 5,2 | Acidobacteria | Solibacteres | Solibacterales | Solibacteraceae (Subgroup 3) | unknown genus | unknown species |
| | OTU106 | 0 | 1,3 ± 2,3 | 42,3 ± 12,7 | 138,3 ± 22,2 | 71 ± 20,7 | 5,1 | Proteobacteria | Gammaproteobacteria | Xanthomonadales | Xanthomonadales Incertae Sedis | Acidibacter | unknown species |
| | OTU356 | 0 | 0 | 22,3 ± 8,1 | 43 ± 7,9 | 22 ± 7,9 | 5,1 | Planctomycetes | Phycisphaerae | Tepidisphaerales | Tepidisphaeraceae | unknown genus | unknown species |
| | OTU1856 | 0 | 0 | 6,3 ± 1,1 | 6,7 ± 0,6 | 3,3 ± 1,5 | 5 | Acidobacteria | Holophagae | Subgroup 7 | unknown family | unknown genus | unknown species |
| | OTU630 | 0,3 ± 0,6 | 0 | 14,3 ± 2,5 | 23,7 ± 3 | 11,7 ± 1,5 | 4,9 | Acidobacteria | Solibacteres | Solibacterales | Solibacteraceae (Subgroup 3) | Candidatus Solibacter | unknown species |
| | OTU1023 | 0 | 0 | 6,3 ± 1,5 | 19 ± 3 | 9,3 ± 4,7 | 4,9 | Proteobacteria | Gammaproteobacteria | Xanthomonadales | Xanthomonadales Incertae Sedis | Acidibacter | unknown species |
| | OTU94 | 1 ± 1 | 0,3 ± 0,6 | 67,3 ± 20,5 | 111,7 ± 9,5 | 54,3 ± 13,6 | 4,9 | Acidobacteria | Blastocatellia | Blastocatellales | Blastocatellaceae (Subgroup 4) | unknown genus | unknown species |
| | OTU907 | 0 | 0 | 9 ± 2,6 | 19,3 ± 2,3 | 9,3 ± 4,2 | 4,8 | Planctomycetes | Planctomycetacia | Planctomycetales | Planctomycetaceae | unknown genus | unknown species |
| | OTU864 | 0 | 0 | 8,7 ± 5,5 | 22,3 ± 2,1 | 10,7 ± 4 | 4,8 | Chlamydiae | Chlamydiae | Chlamydiales | Parachlamydiaceae | Candidatus Metachlamydia | Candidatus Metachlamydia lacustris |
| | OTU1269 | 0 | 0,3 ± 0,6 | 3,7 ± 2,1 | 34,3 ± 10,2 | 16,3 ± 5,5 | 4,8 | Proteobacteria | Gammaproteobacteria | Legionellales | Coxiellaceae | Aquicella | unknown species |
| | OTU456 | 0 | 0 | 5 ± 7,8 | 46,3 ± 10 | 22 ± 10 | 4,7 | Proteobacteria | Gammaproteobacteria | BD72BR169 | unknown family | unknown genus | unknown species |
| | OTU2776 | 0 | 0 | 0,7 ± 0,6 | 5,7 ± 1,1 | 2,7 ± 1,5 | 4,7 | Bacteroidetes | Sphingobacteriia | Sphingobacteriales | unknown family | unknown genus | unknown species |
| | OTU265 | 1,7 ± 2,1 | 0,3 ± 0,6 | 44 ± 9 | 54,7 ± 7 | 25,7 ± 10,1 | 4,7 | Proteobacteria | Gammaproteobacteria | Xanthomonadales | unknown family | unknown genus | unknown species |
| | OTU134 | 2,7 ± 2,5 | 0 | 41,7 ± 19,2 | 161,3 ± 21,7 | 74,7 ± 12,7 | 4,6 | Planctomycetes | Planctomycetacia | Planctomycetales | Planctomycetaceae | unknown genus | unknown species |
| | OTU932 | 0 | 0 | 6 ± 4 | 20,7 ± 3 | 9,3 ± 2,5 | 4,5 | Planctomycetes | OM190 | unknown order | unknown family | unknown genus | unknown species |
| | OTU107 | 0,7 ± 1,1 | 1 ± 1,7 | 51,3 ± 28 | 114,7 ± 3,8 | 51,7 ± 16,6 | 4,5 | Verrucomicrobia | Spartobacteria | Chthoniobacterales | Chthoniobacteraceae | Chthoniobacter | unknown species |
| | OTU343 | 1 ± 1,7 | 0,7 ± 0,6 | 30,3 ± 8,9 | 46 ± 7,5 | 20,7 ± 4,6 | 4,5 | Acidobacteria | Acidobacteria | Acidobacteriales | Acidobacteriaceae (Subgroup 1) | unknown genus | unknown species |
| | OTU170 | 1,3 ± 1,5 | 1 ± 1,7 | 88,7 ± 9,1 | 139,7 ± 12,5 | 62,3 ± 8,1 | 4,5 | Proteobacteria | Gammaproteobacteria | Xanthomonadales | Xanthomonadales Incertae Sedis | Acidibacter | unknown species |
| | OTU383 | 0 | 0 | 48,3 ± 5 | 35 ± 5,2 | 15,3 ± 4,9 | 4,4 | Acidobacteria | Holophagae | Subgroup 7 | unknown family | unknown genus | unknown species |
| | OTU2574 | 0 | 0 | 0 | 7,7 ± 1,1 | 3,3 ± 0,6 | 4,3 | Bacteroidetes | Sphingobacteriia | Sphingobacteriales | Chitinophagaceae | Terrimonas | unknown species |
| | OTU261 | 0 | 1,3 ± 1,5 | 37,3 ± 12,7 | 59,3 ± 14,3 | 25,7 ± 6,1 | 4,3 | Acidobacteria | Subgroup 6 | unknown order | unknown family | unknown genus | unknown species |
| | OTU141 | 1,7 ± 0,6 | 1,3 ± 2,3 | 59,7 ± 22 | 78 ± 8,5 | 33,7 ± 11,7 | 4,3 | Latescibacteria | unknown class | unknown order | unknown family | unknown genus | unknown species |
| | OTU600 | 0 | 0,3 ± 0,6 | 24,7 ± 7,5 | 23,7 ± 2,1 | 10 ± 5 | 4,2 | Acidobacteria | Subgroup 6 | unknown order | unknown family | unknown genus | unknown species |
| | OTU398 | 0 | 0 | 11,3 ± 6,3 | 46,7 ± 14 | 19,7 ± 7,1 | 4,2 | Gemmatimonadetes | Gemmatimonadetes | Gemmatimonadales | Gemmatimonadaceae | Gemmatimonas | unknown species |
| | OTU483 | 0,7 ± 1,1 | 0 | 26,7 ± 4,2 | 28,7 ± 7,8 | 12 ± 6,6 | 4,2 | Parcubacteria | Candidatus Uhrbacteria | unknown order | unknown family | unknown genus | Candidatus Uhrbacteria bacterium GW2011_GWC2_41_11 |
| | OTU1618 | 0 | 1,7 ± 2,1 | 4,3 ± 4,9 | 16 ± 1,7 | 6,7 ± 5,7 | 4,2 | Verrucomicrobia | Spartobacteria | Chthoniobacterales | Chthoniobacteraceae | Chthoniobacter | unknown species |
| | OTU195 | 0 | 0 | 36 ± 16,6 | 76,3 ± 9 | 31,7 ± 8,5 | 4,1 | Proteobacteria | Gammaproteobacteria | Xanthomonadales | Xanthomonadales Incertae Sedis | Acidibacter | unknown species |
| | OTU2958 | 0 | 0 | 0,3 ± 0,6 | 5,7 ± 0,6 | 2,3 ± 1,1 | 4,1 | Bacteroidetes | Bacteroidetes VC2.1 Bac | unknown order | unknown family | unknown genus | unknown species |
| | OTU81 | 3,3 ± 1,1 | 0 | 80,3 ± 7,4 | 232 ± 6,2 | 95 ± 32,2 | 4,1 | Proteobacteria | Gammaproteobacteria | Xanthomonadales | Xanthomonadales Incertae Sedis | Acidibacter | unknown species |
| | OTU851 | 0 | 0 | 6,3 ± 4 | 27 ±2 | 11 ± 4,6 | 4,1 | Planctomycetes | OM190 | unknown order | unknown family | unknown genus | unknown species |
| | OTU786 | 0,3 ± 0,6 | 0 | 9 ± 3,5 | 22,3 ± 2,1 | 9 ± 1 | 4 | Proteobacteria | Betaproteobacteria | Nitrosomonadales | Nitrosomonadaceae | unknown genus | unknown species |
| | OTU245 | 3,3 ± 4,2 | 0 | 3 ± 1 | 82 ± 15,39 | 32,7 ± 14,5 | 4 | Actinobacteria | Actinobacteria | Streptomycetales | Streptomycetaceae | unknown genus | unknown species |
| | OTU295 | 0 | 0,7 ± 1,1 | 36,3 ± 4,6 | 48,7 ± 14,6 | 19 ± 6,6 | 3,9 | Acidobacteria | Subgroup 6 | unknown order | unknown family | unknown genus | unknown species |
| | OTU2225 | 0 | 0,3 ± 0,6 | 2,7 ± 0,6 | 6 ± 1 | 2,3 ± 1,1 | 3,9 | Proteobacteria | Deltaproteobacteria | Myxococcales | Haliangiaceae | Haliangium | unknown species |
| | OTU837 | 0 | 0 | 17,7 ± 6,5 | 20 ± 1 | 7,7 ± 2,9 | 3,8 | Acidobacteria | Acidobacteria | Acidobacteriales | Acidobacteriaceae (Subgroup 1) | unknown genus | unknown species |
| | OTU101 | 0,7 ± 1,1 | 0,7 ± 1,1 | 63 ± 39,3 | 117,3 ± 8 | 44 ± 7,8 | 3,8 | Chlamydiae | Chlamydiae | Chlamydiales | Parachlamydiaceae | Neochlamydia | unknown species |
| | OTU3222 | 0 | 0 | 2 ± 1 | 5,3 ± 0,6 | 2 ± 1,7 | 3,8 | Proteobacteria | Deltaproteobacteria | Desulfurellales | Desulfurellaceae | H16 | unknown species |
| | OTU644 | 1,3 ± 2,3 | 0,3 ± 0,6 | 22,3 ± 5,5 | 28,7 ± 4,7 | 1067 ± 4,2 | 3,7 | Gemmatimonadetes | Gemmatimonadetes | Gemmatimonadales | Gemmatimonadaceae | Gemmatimonas | unknown species |
| | OTU93 | 1,7 ± 2,9 | 2,7 ± 4,6 | 42,3 ± 20,2 | 245,3 ± 32,1 | 91 ± 8,7 | 3,7 | Proteobacteria | Gammaproteobacteria | Xanthomonadales | unknown family | unknown genus | unknown species |
| | OTU1187 | 0,7 ± 1,1 | 0 | 9,3 ± 5,1 | 22,7 ± 3,8 | 8,3 ± 6,8 | 3,7 | Proteobacteria | Alphaproteobacteria | Caulobacterales | Caulobacteraceae | Phenylobacterium | unknown species |
| | OTU1293 | 0 | 0 | 4,3 ± 2,5 | 21 ± 5 | 7,7 ± 3,8 | 3,7 | Acidobacteria | Blastocatellia | Blastocatellales | Blastocatellaceae (Subgroup 4) | DS-100 | unknown species |
| | OTU315 | 0 | 0 | 34,7 ± 3,5 | 45,7 ± 2,5 | 16,7 ± 7,6 | 3,6 | Planctomycetes | Phycisphaerae | Tepidisphaerales | Tepidisphaeraceae | unknown genus | unknown species |
| | OTU475 | 0 | 0 | 19,3 ± 17,9 | 55 ± 10,5 | 20 ± 1,7 | 3,6 | Proteobacteria | Gammaproteobacteria | Xanthomonadales | unknown family | unknown genus | unknown species |
| | OTU993 | 0 | 0 | 13,7 ± 2,5 | 23 ± 8,5 | 8,3 ± 2,1 | 3,6 | Verrucomicrobia | OPB35 soil group | unknown order | unknown family | unknown genus | unknown species |
| | OTU1551 | 0 | 1 ± 1,7 | 6 ± 3,6 | 17,7 ± 2,9 | 6,3 ± 3 | 3,6 | Cyanobacteria | Melainabacteria | Obscuribacterales | unknown family | unknown genus | unknown species |
| | OTU200 | 0 | 1,3 ± 2,3 | 51 ± 19,7 | 62,3 ± 11,4 | 22,3 ± 9,4 | 3,6 | Planctomycetes | Phycisphaerae | Tepidisphaerales | Tepidisphaeraceae | unknown genus | unknown species |
| | OTU2693 | 0 | 0 | 3,7 ± 3,8 | 11,3 ± 1,1 | 4 ± 1,7 | 3,5 | TM6 (Dependentiae) | unknown class | unknown order | unknown family | unknown genus | unknown species |
| | OTU693 | 0 | 0 | 12,3 ± 1,5 | 23 ±6,6 | 8 ± 2,6 | 3,5 | Proteobacteria | Gammaproteobacteria | Xanthomonadales | Xanthomonadaceae | Tahibacter | unknown species |
| | OTU1073 | 1 ± 1,7 | 0 | 7,7 ± 4 | 15,7 ± 3,5 | 5,3 ± 3 | 3,4 | Acidobacteria | Acidobacteria | Acidobacteriales | Acidobacteriaceae (Subgroup 1) | unknown genus | unknown species |
| | OTU1189 | 0,3 ± 0,6 | 0 | 8,3 ± 4 | 15,7 ± 4 | 5,3 ± 2,5 | 3,4 | Planctomycetes | OM190 | unknown order | unknown family | unknown genus | unknown species |
| | OTU1203 | 0 | 0 | 0,7 ± 1,1 | 19,3 ± 7,8 | 6,3 ± 1,5 | 3,3 | Saccharibacteria | unknown class | unknown order | unknown family | unknown genus | unknown species |
| | OTU1697 | 0 | 0 | 0 | 14,3 ± 5,9 | 4,7 ± 2,9 | 3,3 | Proteobacteria | Deltaproteobacteria | Myxococcales | Blrii41 | unknown genus | unknown species |
| | OTU1004 | 0 | 0 | 7 ± 1 | 19,7 ± 3,2 | 6,3 ± 4,5 | 3,2 | Proteobacteria | Gammaproteobacteria | Xanthomonadales | unknown family | unknown genus | unknown species |
| | OTU1433 | 0 | 0 | 0,3 ± 0,6 | 15,7 ± 3,8 | 5 ± 5 | 3,2 | Actinobacteria | Actinobacteria | Micromonosporales | Micromonosporaceae | Catellatospora | unknown species |
| | OTU174 | 0 | 0 | 19,3 ± 14,6 | 94 ± 17,7 | 30 ± 12,5 | 3,2 | Chlamydiae | Chlamydiae | Chlamydiales | Parachlamydiaceae | Neochlamydia | unknown species |
| | OTU827 | 0 | 0 | 13,7 ± 3,5 | 20 ±5,3 | 6,3 ± 1,5 | 3,2 | Proteobacteria | Alphaproteobacteria | Rhizobiales | Rhizobiales Incertae Sedis | Rhizomicrobium | unknown species |
| | OTU433 | 0,3 ± 0,6 | 0,7 ± 0,6 | 11,7 ± 11,6 | 68 ± 3 | 21 ± 8,7 | 3,1 | Proteobacteria | Deltaproteobacteria | Myxococcales | Blrii41 | unknown genus | unknown species |
| | OTU1543 | 0 | 0,7 ± 1,1 | 5,3 ± 6,7 | 29 ± 9,2 | 8,7 ± 3,5 | 3 | Proteobacteria | Gammaproteobacteria | Xanthomonadales | Xanthomonadales Incertae Sedis | Acidibacter | unknown species |
| Bacteria | OTU365 | 0,3 ± 0,6 | 2,3 ± 4 | 43,7 ± 12 | 82,7 ± 16,5 | 24 ± 3,6 | 2,9 | Proteobacteria | Alphaproteobacteria | Sphingomonadales | Erythrobacteraceae | Altererythrobacter | unknown species |
| | OTU432 | 0 | 0,7 ± 1,1 | 11 ± 9,9 | 72,3 ± 10 | 20,7 ± 3,8 | 2,9 | Proteobacteria | Deltaproteobacteria | Myxococcales | Blrii41 | unknown genus | unknown species |
| | OTU697 | 0 | 0,3 ± 0,6 | 1 ± 1,7 | 35 ± 9,5 | 10 ± 5,6 | 2,9 | Actinobacteria | Actinobacteria | Micromonosporales | Micromonosporaceae | Rhizocola | unknown species |
| | OTU345 | 0,3 ± 0,6 | 2 ±3,5 | 60 ± 20,8 | 56,3 ± 7,6 | 16 ± 3 | 2,8 | Planctomycetes | Phycisphaerae | CPla-3 termite group | unknown family | unknown genus | unknown species |
| | OTU658 | 0 | 0 | 24 ± 4 | 48,7 ± 7,5 | 13,7 ± 1,5 | 2,8 | Gemmatimonadetes | Gemmatimonadetes | Gemmatimonadales | Gemmatimonadaceae | Gemmatimonas | unknown species |
| | OTU1056 | 0 | 0 | 10 ± 2,6 | 19 ± 3,6 | 5,3 ± 1,5 | 2,8 | Proteobacteria | Betaproteobacteria | Burkholderiales | Comamonadaceae | Ideonella | unknown species |
| | OTU339 | 0 | 0 | 37,7 ± 4 | 42,7 ± 12,9 | 11,7 ± 4,7 | 2,7 | Acidobacteria | Subgroup 6 | unknown order | unknown family | unknown genus | unknown species |
| | OTU1760 | 0 | 0 | 4 ± 3 | 29,7 ± 6,7 | 8 ± 3,5 | 2,7 | Chloroflexi | Anaerolineae | Anaerolineales | Anaerolineaceae | unknown genus | unknown species |
| | OTU162 | 1,7 ± 2,9 | 0,7 ± 1,1 | 50,3 ± 32,5 | 81,7 ± 12,3 | 21,7 ± 8,1 | 2,7 | Acidobacteria | Solibacteres | Solibacterales | Solibacteraceae (Subgroup 3) | Bryobacter | unknown species |
| | OTU797 | 0 | 0,7 ± 0,6 | 0,7 ± 1,1 | 28,3 ± 2,1 | 7,3 ± 2,5 | 2,6 | Saccharibacteria | unknown class | unknown order | unknown family | unknown genus | unknown species |
| | OTU2141 | 0 | 0 | 3,3 ± 3 | 13 ± 4,6 | 3,3 ± 1,5 | 2,6 | Proteobacteria | Deltaproteobacteria | Myxococcales | Haliangiaceae | Haliangium | unknown species |
| | OTU1423 | 0 | 0,3 ± 0,6 | 14 ± 1 | 21 ± 7,6 | 5,3 ± 2,5 | 2,5 | Acidobacteria | Acidobacteria | Acidobacteriales | Acidobacteriaceae (Subgroup 1) | unknown genus | bacterium Ellin6547 |
| | OTU273 | 0,3 ± 0,6 | 1,3 ± 2,3 | 79 ± 24,5 | 75,3 ± 8,1 | 19 ± 12,5 | 2,5 | Acidobacteria | Solibacteres | Solibacterales | Solibacteraceae (Subgroup 3) | Bryobacter | unknown species |
| | OTU2515 | 0 | 0 | 2 ± 1,7 | 8 ± 1,7 | 2 ± 1 | 2,5 | Planctomycetes | Pla4 lineage | unknown order | unknown family | unknown genus | unknown species |
| | OTU1848 | 0 | 0,3 ± 0,6 | 1,3 ± 1,1 | 10,7 ± 0,6 | 2,7 ± 1,1 | 2,5 | FCPU426 | unknown class | unknown order | unknown family | unknown genus | unknown species |
| | OTU3296 | 0 | 0 | 1 ± 1 | 5,3 ± 1,5 | 1,3 ± 0,6 | 2,5 | Proteobacteria | Deltaproteobacteria | Myxococcales | Sandaracinaceae | Sandaracinus | unknown species |
| | OTU1365 | 0 | 0 | 0 | 17,7 ± 5,7 | 4,3 ± 2,3 | 2,5 | Proteobacteria | Deltaproteobacteria | Myxococcales | Blrii41 | unknown genus | unknown species |
| | OTU586 | 0 | 0,7 ± 1,1 | 16,3 ± 11,5 | 29,7 ± 5,7 | 7 ± 4,4 | 2,4 | Planctomycetes | Phycisphaerae | Tepidisphaerales | Tepidisphaeraceae | unknown genus | unknown species |
| | OTU1481 | 0 | 0 | 8,7 ± 2,3 | 10 ± 2,6 | 2,3 ± 1,5 | 2,3 | Planctomycetes | OM190 | unknown order | unknown family | unknown genus | unknown species |
| | OTU633 | 0 | 0 | 18 ± 7,5 | 24,3 ± 4 | 5,7 ± 1,1 | 2,3 | Acidobacteria | Subgroup 6 | unknown order | unknown family | unknown genus | unknown species |
| | OTU1566 | 0,3 ± 0,6 | 0,3 ± 0,6 | 0,7 ± 1,1 | 14,3 ± 4,2 | 3,3 ± 0,6 | 2,3 | Proteobacteria | Deltaproteobacteria | Myxococcales | Phaselicystidaceae | Phaselicystis | unknown species |
| | OTU1024 | 0 | 0,7 ± 1,1 | 28,3 ± 2,5 | 23 ± 7,2 | 5,3 ± 1,5 | 2,3 | Proteobacteria | Gammaproteobacteria | Xanthomonadales | Xanthomonadaceae | Rhodanobacter | unknown species |
| | OTU515 | 0,3 ± 0,6 | 0,3 ± 0,6 | 11,7 ± 4 | 40,7 ± 2,1 | 9,3 ± 2,3 | 2,3 | Acidobacteria | Acidobacteria | Acidobacteriales | Acidobacteriaceae (Subgroup 1) | Candidatus Koribacter | unknown species |
| | OTU227 | 0,7 ± 0,6 | 0 | 83,7 ± 9,1 | 84,3 ± 5,9 | 19,3 ± 1,5 | 2,3 | Proteobacteria | Gammaproteobacteria | Xanthomonadales | Xanthomonadaceae | unknown genus | unknown species |
| | OTU387 | 0 | 0,3 ± 0,6 | 33,7 ± 12,2 | 43 ± 5,3 | 9,3 ± 1,5 | 2,2 | Proteobacteria | Gammaproteobacteria | Xanthomonadales | Xanthomonadaceae | Dokdonella | Dokdonella soli |
| | OTU1564 | 0 | 0 | 3,7 ± 3,5 | 11,3 ± 1,5 | 2,3 ± 1,5 | 2,1 | Bacteroidetes | Sphingobacteriia | Sphingobacteriales | Chitinophagaceae | unknown genus | unknown species |
| | OTU1609 | 0 | 0 | 1,7 ± 0,6 | 14,7 ± 3,8 | 3 ±1 | 2 | Proteobacteria | Deltaproteobacteria | Myxococcales | Haliangiaceae | Haliangium | unknown species |
| | OTU530 | 1,3 ± 2,3 | 0,3 ± 0,6 | 14,7 ± 3,5 | 52,7 ± 4,6 | 10,7 ± 2,5 | 2 | Proteobacteria | Gammaproteobacteria | Xanthomonadales | Xanthomonadaceae | unknown genus | unknown species |
| | OTU1677 | 0 | 0 | 6,3 ± 1,1 | 10,3 ± 1,1 | 2 ± 1,7 | 1,9 | Acidobacteria | Holophagae | Subgroup 7 | unknown family | unknown genus | unknown species |
| | OTU1200 | 0 | 0,3 ± 0,6 | 9 ± 3 | 29,3 ± 4,7 | 5,7 ± 4,2 | 1,9 | Verrucomicrobia | Opitutae | Opitutales | Opitutaceae | Opitutus | unknown species |
| | OTU906 | 0 | 0 | 11,7 ± 3 | 19,3 ± 4,7 | 3,7 ± 1,1 | 1,9 | Acidobacteria | Acidobacteria | Acidobacteriales | Acidobacteriaceae (Subgroup 1) | unknown genus | unknown species |
| | OTU2681 | 0 | 0 | 0,3 ± 0,6 | 9 ± 4 | 1,7 ± 1,1 | 1,9 | Proteobacteria | Gammaproteobacteria | Xanthomonadales | unknown family | unknown genus | unknown species |
| | OTU1392 | 0 | 0 | 1,7 ± 2,1 | 17 ± 8,9 | 3 ± 2 | 1,8 | Planctomycetes | OM190 | unknown order | unknown family | unknown genus | unknown species |
| | OTU1904 | 0 | 0,3 ± 0,6 | 4,7 ± 0,6 | 7,7 ± 3,5 | 1,3 ± 0,6 | 1,7 | Planctomycetes | Pla3 lineage | unknown order | unknown family | unknown genus | unknown species |
| | OTU1951 | 0 | 0 | 3,7 ± 4 | 17,3 ± 1,1 | 3 ±1,7 | 1,7 | Acidobacteria | Acidobacteria | Acidobacteriales | Acidobacteriaceae (Subgroup 1) | unknown genus | unknown species |
| | OTU2612 | 0 | 0 | 3 ± 2,6 | 11,7 ± 0,6 | 2 ± 1,7 | 1,7 | Planctomycetes | Phycisphaerae | Tepidisphaerales | Tepidisphaeraceae | unknown genus | unknown species |
| | OTU891 | 0 | 0 | 6,3 ± 5,6 | 24,3 ± 9,3 | 4 ± 3,6 | 1,6 | Proteobacteria | Gammaproteobacteria | Xanthomonadales | unknown family | unknown genus | unknown species |
| | OTU737 | 0 | 0,7 ± 1,1 | 15,3 ± 21,4 | 49,7 ± 9,4 | 8 ± 3,6 | 1,6 | Proteobacteria | Deltaproteobacteria | Myxococcales | Blrii41 | unknown genus | unknown species |
| | OTU1749 | 0 | 0 | 7,3 ± 1,5 | 10,7 ± 3,1 | 1,7 ± 1,1 | 1,6 | Proteobacteria | Deltaproteobacteria | Myxococcales | Haliangiaceae | Haliangium | unknown species |
| | OTU146 | 0,7 ± 0,6 | 1 ± 1,7 | 48 ± 4,6 | 109,3 ± 9,2 | 17 ± 5 | 1,6 | Proteobacteria | Gammaproteobacteria | Xanthomonadales | Xanthomonadaceae | unknown genus | unknown species |
| | OTU1381 | 0 | 0,3 ± 0,6 | 10,7 ± 2,5 | 13 ± 4,6 | 2 ± 1 | 1,5 | Acidobacteria | Acidobacteria | Acidobacteriales | Acidobacteriaceae (Subgroup 1) | unknown genus | unknown species |
| | OTU239 | 0,7 ± 1,1 | 2,3 ± 4 | 51,3 ± 23,4 | 76,3 ± 32,6 | 11,3 ± 6,4 | 1,5 | Verrucomicrobia | Spartobacteria | Chthoniobacterales | Chthoniobacterales Incertae Sedis | Terrimicrobium | unknown species |
| | OTU354 | 0 | 0,3 ± 0,6 | 37,7 ± 4 | 51 ±7 | 6 ± 1 | 1,2 | Verrucomicrobia | OPB35 soil group | unknown order | unknown family | unknown genus | unknown species |
| | OTU1588 | 0 | 0 | 4,3 ± 4,9 | 14,3 ± 2,3 | 1,7 ± 1,1 | 1,2 | Proteobacteria | Gammaproteobacteria | Xanthomonadales | Xanthomonadaceae | Panacagrimonas | unknown species |
| | OTU716 | 0 | 0 | 3,7 ± 2,5 | 39,7 ± 12 | 4,3 ± 1,5 | 1,1 | Spirochaetae | Spirochaetes | Spirochaetales | Spirochaetaceae | Spirochaeta 2 | unknown species |
| | OTU380 | 0 | 0 | 30 ± 1,7 | 52,3 ± 9 | 5,7 ± 6,4 | 1,1 | Acidobacteria | Acidobacteria | Acidobacteriales | Acidobacteriaceae (Subgroup 1) | unknown genus | unknown species |
| | OTU1046 | 0 | 0 | 6,3 ± 6,1 | 22 ± 7 | 2,3 ± 1,5 | 1,1 | Bacteroidetes | Sphingobacteriia | Sphingobacteriales | Sphingobacteriaceae | Mucilaginibacter | unknown species |
| | OTU88 | 1 ± 1,7 | 1,3 ± 2,3 | 6 ± 5,6 | 217 ± 53,6 | 17 ± 4,4 | 0,8 | Actinobacteria | Actinobacteria | Micromonosporales | Micromonosporaceae | Hamadaea | unknown species |
| | OTU212 | 0,3 ± 0,6 | 0 | 22 ± 14,1 | 129,7 ± 33,9 | 8,7 ± 7,4 | 0,7 | Proteobacteria | Deltaproteobacteria | Myxococcales | Haliangiaceae | Haliangium | unknown species |
| | OTU406 | 0 | 0 | 0,3 ± 0,6 | 70,7 ± 38,8 | 4,3 ± 1,1 | 0,6 | Proteobacteria | Gammaproteobacteria | Legionellales | Coxiellaceae | Aquicella | unknown species |
| Fungi | OTU1919 | 2 ± 3,5 | 0,3 ± 0,6 | 50,7 ± 28,4 | 481,7 ± 114,5 | 284,7 ± 48,2 | 1,7 | Ascomycota | Sordariomycetes | Sordariales | Lasiosphaeriaceae | Cladorrhinum | Cladorrhinum bulbillosum |
| | OTU2013 | 5 ± 2,6 | 1,3 ± 2,3 | 94 ± 27,6 | 763,3 ± 188,4 | 406 ± 67 | 1,9 | Ascomycota | Sordariomycetes | Sordariales | Unknown | Unknown | Unknown |
| | OTU2172 | 3,3 ± 5,8 | 0 | 17,7± 5,7 | 183,7 ± 19,2 | 74,3 ± 18,7 | 2,5 | Ascomycota | Sordariomycetes | Sordariales | Chaetomiaceae | Chaetomium | Unknown |
| | OTU2181 | 0,3 ± 0,6 | 0 | 17,7 ± 3,2 | 77,3 ± 29,3 | 24 ± 17,6 | 3,2 | Ascomycota | Unknown | Unknown | Unknown | Unknown | Unknown |
| | OTU1985 | 2,7 ± 4,6 | 0 | 7,3 ± 7,1 | 63,7 ± 17,9 | 16 ± 6,1 | 4 | Unknown | Unknown | Unknown | Unknown | Unknown | Unknown |
| | OTU2147 | 0 | 0 | 3 ± 4,1 | 27,3 ± 11 | 5,3 ± 4,5 | 5,1 | Ascomycota | Sordariomycetes | Sordariales | Lasiosphaeriaceae | Unknown | Unknown |
| | OTU2144 | 0 | 0 | 6,3 ± 1,5 | 65,3 ± 19,3 | 12 ± 2,6 | 5,4 | Ascomycota | Unknown | Unknown | Unknown | Unknown | Unknown |
| | OTU2836 | 4 ± 6,9 | 0 | 75,3 ± 31,2 | 184 ± 54,8 | 22,3 ± 16,1 | 8,2 | Unknown | Unknown | Unknown | Unknown | Unknown | Unknown |
| | OTU652 | 0 | 0,3 ± 0,6 | 2,3 ± 3,2 | 35 ± 13 | 2,3 ± 3,2 | 15 | Basidiomycota | Agaricomycetes | Agaricales | Unknown | Unknown | Unknown |
| | OTU3234 | 886 ± 242,9 | 55 ± 7 | 0 | 1 ± 0 | 0 | - | Basidiomycota | Tremellomycetes | Tremellales | Tremellaceae | Cryptococcus | Cryptococcus wieringae |
