# Supplementary figures and images for: Specific recruitment of soil bacteria and fungi decomposers following a biostimulant application increased crop residues mineralization
Source: PLoS One. 2018 Dec 31;13(12):e0209089. doi: 10.1371/journal.pone.0209089 (PMC6312294; doi:10.1371/journal.pone.0209089)

A) Bacteria

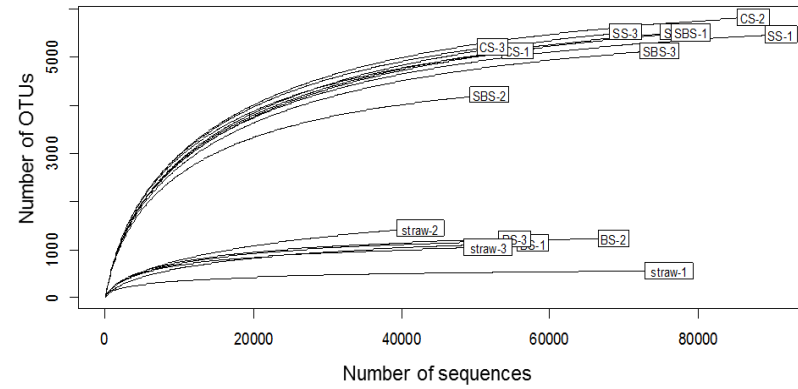

B) Fungi

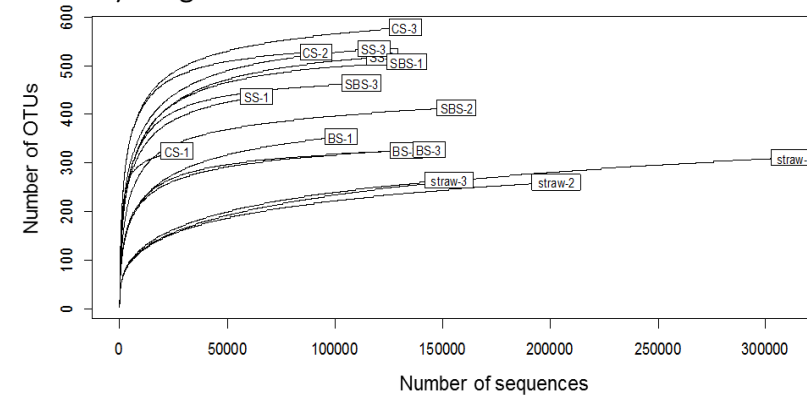

C) Archaea

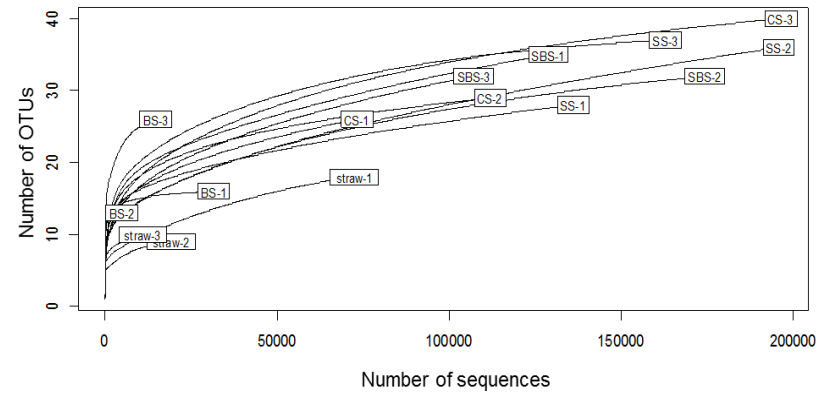

Supplement: S1 Fig — BS: biostimulant samples, CS: control soil, SS: soil with straw, SBS: soil with straw and BS. (PDF) [file pone.0209089.s004.pdf]

A) Bacteria

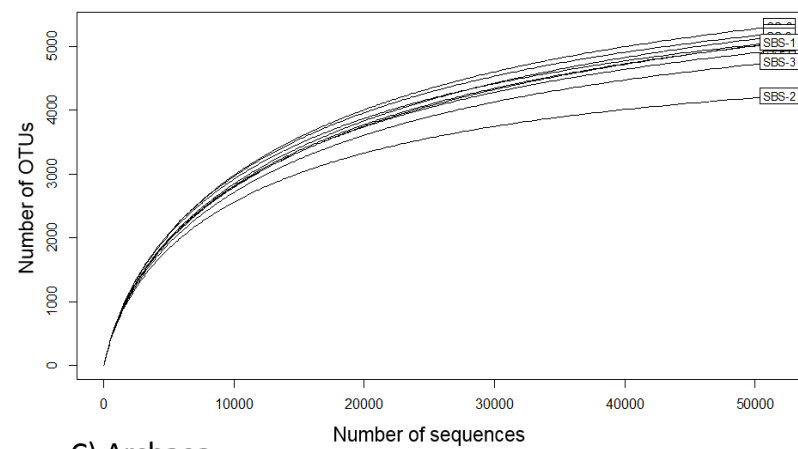

B) Fungi

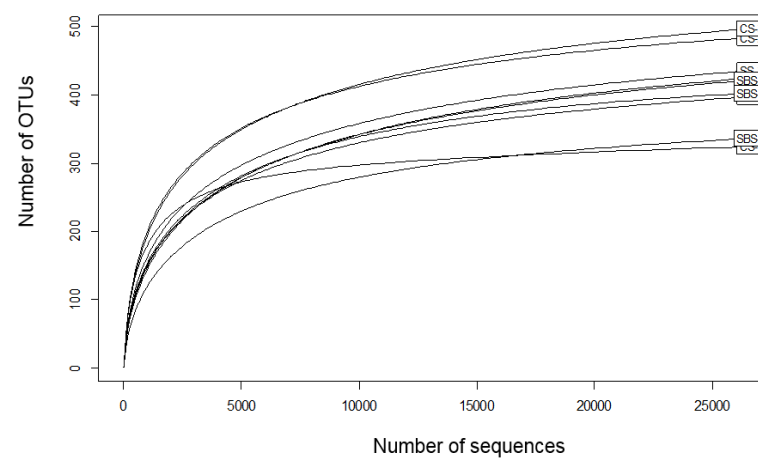

C) Archaea

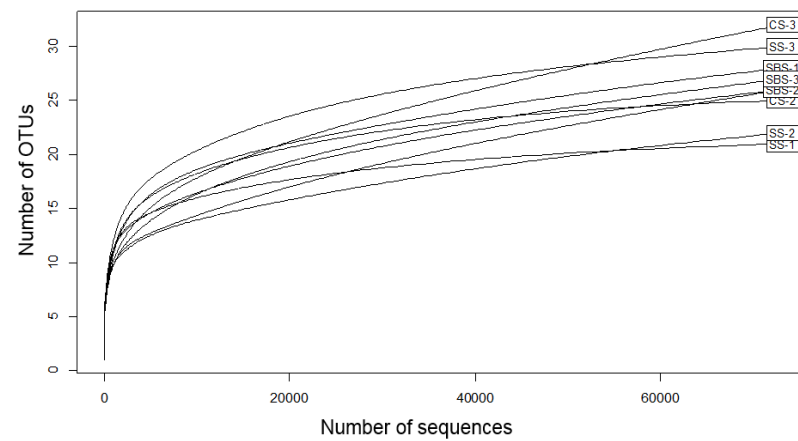

Supplement: S2 Fig — (PDF) [file pone.0209089.s005.pdf]

A) Bacteria

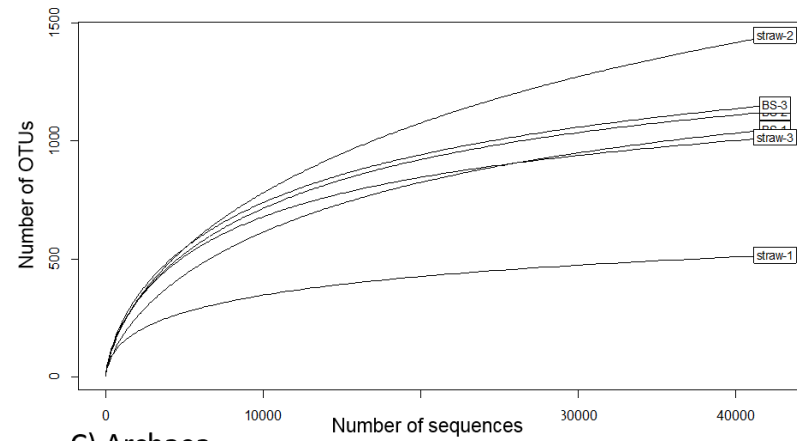

B) Fungi

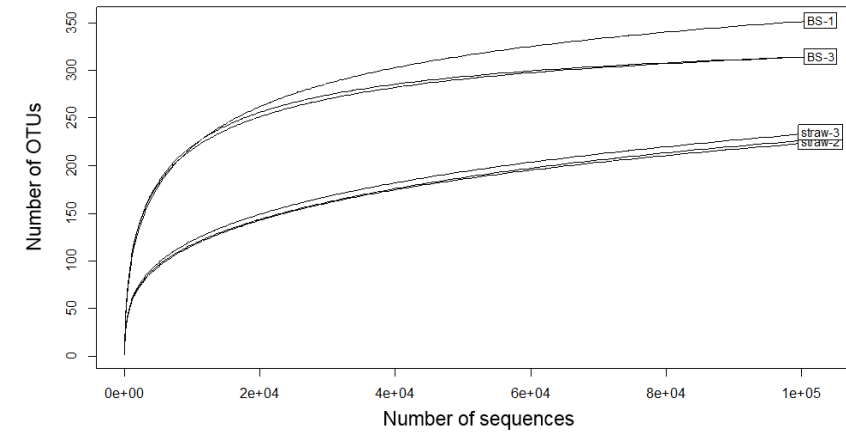

C) Archaea

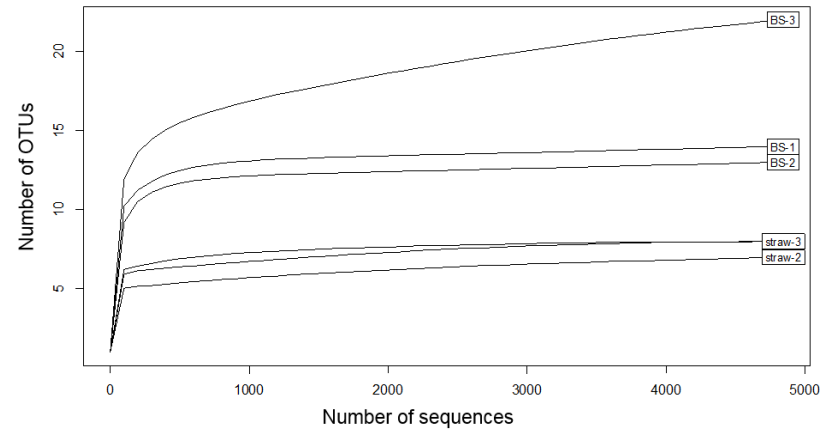

Supplement: S3 Fig — (PDF) [file pone.0209089.s006.pdf]
